# Oscillatory Behavior of Neutrophils under Opposing Chemoattractant Gradients Supports a Winner-Take-All Mechanism

**DOI:** 10.1371/journal.pone.0085726

**Published:** 2014-01-21

**Authors:** Matthew B. Byrne, Yuki Kimura, Ashish Kapoor, Yuan He, Kewin S. Mattam, Katherine M. Hasan, Luke N. Olson, Fei Wang, Paul J. A. Kenis, Christopher V. Rao

**Affiliations:** 1 Department of Chemical and Biomolecular Engineering, University of Illinois at Urbana-Champaign, Urbana, Illinois, United States of America; 2 Department of Cell and Developmental Biology, University of Illinois at Urbana-Champaign, Urbana, Illinois, United States of America; 3 Department of Computer Science, University of Illinois at Urbana-Champaign, Urbana, Illinois, United States of America; 4 Department of Pathology, University of Michigan, Ann Arbor, Michigan, United States of America; Fondazione Edmund Mach, Research and Innovation Centre, Italy

## Abstract

Neutrophils constitute the largest class of white blood cells and are the first responders in the innate immune response. They are able to sense and migrate up concentration gradients of chemoattractants in search of primary sites of infection and inflammation through a process known as chemotaxis. These chemoattractants include formylated peptides and various chemokines. While much is known about chemotaxis to individual chemoattractants, far less is known about chemotaxis towards many. Previous studies have shown that in opposing gradients of intermediate chemoattractants (interleukin-8 and leukotriene B_4_), neutrophils preferentially migrate toward the more distant source. In this work, we investigated neutrophil chemotaxis in opposing gradients of chemoattractants using a microfluidic platform. We found that primary neutrophils exhibit oscillatory motion in opposing gradients of intermediate chemoattractants. To understand this behavior, we constructed a mathematical model of neutrophil chemotaxis. Our results suggest that sensory adaptation alone cannot explain the observed oscillatory motion. Rather, our model suggests that neutrophils employ a winner-take-all mechanism that enables them to transiently lock onto sensed targets and continuously switch between the intermediate attractant sources as they are encountered. These findings uncover a previously unseen behavior of neutrophils in opposing gradients of chemoattractants that will further aid in our understanding of neutrophil chemotaxis and the innate immune response. In addition, we propose a winner-take-all mechanism allows the cells to avoid stagnation near local chemical maxima when migrating through a network of chemoattractant sources.

## Introduction

Neutrophil chemotaxis plays a prominent role in the innate immune response [1]–[3]. A number of chemical signals are produced at sites of infection or inflammation and then diffuse into the surrounding tissue [4], [5]. Neutrophils sense these chemoattractants and move in the direction where their concentration is greatest, thereby locating the source of the chemoattractants and their associated targets. Neutrophils respond to many different chemoattractants including: (i) formyl-methionylleucylphenylalanine (fMLP) secreted by the infecting microbes [6]–[8]; (ii) chemokines such as interleukin-8 (IL-8), growth-related gene product α (GROα), leukotriene B_4_ (LTB_4_), and stromal cell-derived factor 1 (SDF-1) secreted by endothelial cells, mast cells, monocytes, and also by neutrophils themselves [9]–[16]; (iii) a glycoprotein fragment, C5a, produced by the complement system [17], [18]; and (iv) hydrogen peroxide, produced by damaged tissue [19], [20]. Each one of these chemoattractants is able to elicit directed cell migration. However, when homing in on their targets, neutrophils are confronted with a complex array of these chemoattractants emanating from multiple sources. For instance, neutrophils encounter intermediate chemoattractants, such as IL-8 and LTB_4_, on the surface of the endothelium and adhere [21]–[23]. There, the cells are presented with additional chemoattractant gradients and must migrate away from these initial chemoattractants toward the source of other chemoattractants. Clearly, neutrophils need to distinguish between these various signals and employ some sort of logic to prioritize among them.

Previous studies have shown that neutrophils selectively migrate toward end-target chemoattractants such as fMLP and C5a even when opposing gradients of endogenous, intermediate, chemoattractants are present [24]–[26]. These results demonstrate that neutrophils discriminate between chemoattractants and will preferentially migrate toward those produced proximal to sites of infection. The logic is less clear when neutrophils are confronted with competing gradients of intermediate chemoattractants. Foxman and coworkers, for example, found that when confronted with opposing gradients of IL-8 and LTB_4_, neutrophils tended to migrate toward the more distant attractant source and away from the more proximal one, independent of the chemoattractant species [25]. They hypothesized that such a mechanism enables neutrophils to navigate stepwise through sequential fields of intermediate chemoattractants while homing in on their end target. Meanwhile, others have utilized microfluidic devices to study neutrophil migration in opposing IL-8 and LTB_4_ gradients [24], [27], [28]. These efforts have focused particularly on the prioritization between these chemicals in the short term, such as whether LTB_4_ can influence chemotaxis towards IL-8.

While the mechanism for the signaling hierarchy between chemoattractants is not known, current results suggest that the two classes operate along different signal transduction pathways altogether - in particular, chemotaxis to the end-target attractants fMLP and C5a involves the p38 mitogen-activated protein kinase (p38 MAPK) pathway, whereas chemotaxis towards IL-8, LTB_4_, and MIP-2 likely involves the phosphatidylinositol-3-OH (PI3K)/phosphatase and tensin homolog (PTEN) pathway [8], [24], [29]. The crosstalk between these pathways is thought to involve PTEN, a known PI3K antagonist, via p38 MAPK-mediated recruitment to the cell circumference [26], [30]. Consequently, in the presence of any end-target chemoattractant, chemotaxis toward the intermediary attractants is suppressed [24], [25], [28], [31].

In this work, we investigated neutrophil chemotaxis in opposing linear gradients of chemoattractants using microfluidic devices. Extending the results of Foxman and coworkers, we demonstrate that neutrophils will migrate back and forth in oscillatory manner when confronted with opposing gradients of IL-8 and LTB_4_. Based on these results, we developed a mathematical model of neutrophil chemotaxis. Our modeling results and associated analysis demonstrates that basic sensory adaptation alone cannot explain the oscillatory migration patterns observed in our experiments. Rather, our results support a model where cells reversibly lock on and off different chemoattractant signals. Using this model, we show how this mechanism allows neutrophils to locate the sites of infection in the face of complex chemoattractant cues.

## Results

### Chemotaxis in single chemoattractant gradients

**Figure 1:**
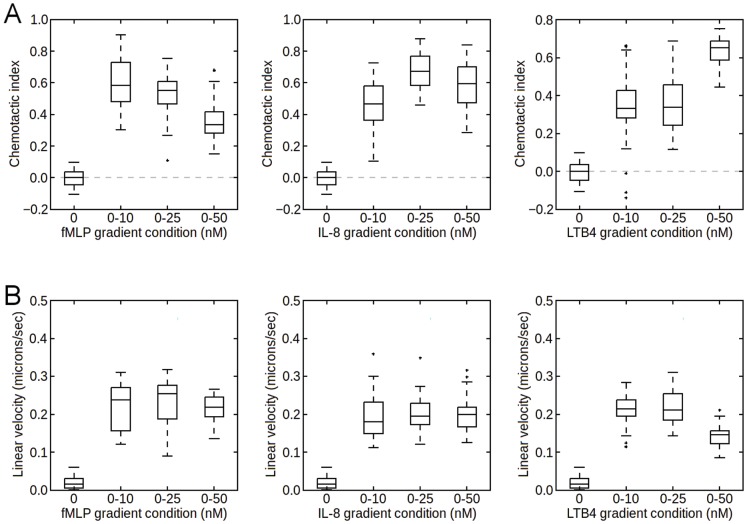
Chemotaxis in single attractant gradients. [A] Chemotactic index in single attractant gradients. [B] Average linear velocities of the cells from the single attractant gradient experiments. In each experiment, 30 cells were tracked for 20 minutes.

We first analyzed chemotaxis toward single gradients of fMLP, IL-8, and LTB_4_ by measuring the chemotactic index and average linear velocities using a microfluidic platform [13]. In the microfluidic platform, the concentration profiles established were linear across the channel width with small deviations from linearity at each end of the channel, as can be seen in Figure S1. In each case, linear concentration profiles of 0–10 nM, 0–25 nM and 0–50 nM were applied across the 350 µm channel, with no gradient as the control. These conditions were chosen roughly for their ability to produce optimal chemotaxis. As evidenced by the positive mean chemotactic indices in Figure 1, most cells migrated up each chemoattractant gradient as expected. However, in each case, the mean response varied according to the gradient condition with no obvious trend. While the chemotactic index has previously been shown to correlate weakly with the slope of the gradient [25], [32], we found that the cells were most responsive to fMLP in the 0–10 or 0–25 nM range. For IL-8, cells responded optimally to the 0–25 nM gradient. Finally, for LTB_4_, cells were most responsive to the 0–50 nM gradient. The linear velocities of these cells, however, did not exhibit any trends. In particular, the linear velocity does not correlated with the slope of the gradient. In addition, we analyzed cell migration in isotropic environments (Figure S2). We found that cells exhibit motion resembling persistent random walk as seen previously [33]. Overall, the chemotactic responses toward the single chemoattractant gradients were similar to previous reports [24], [25], [27], [28], [31], [32], [34], [35].

### Response to single gradients superimposed over an alternate isotropic attractant field

**Figure 2:**
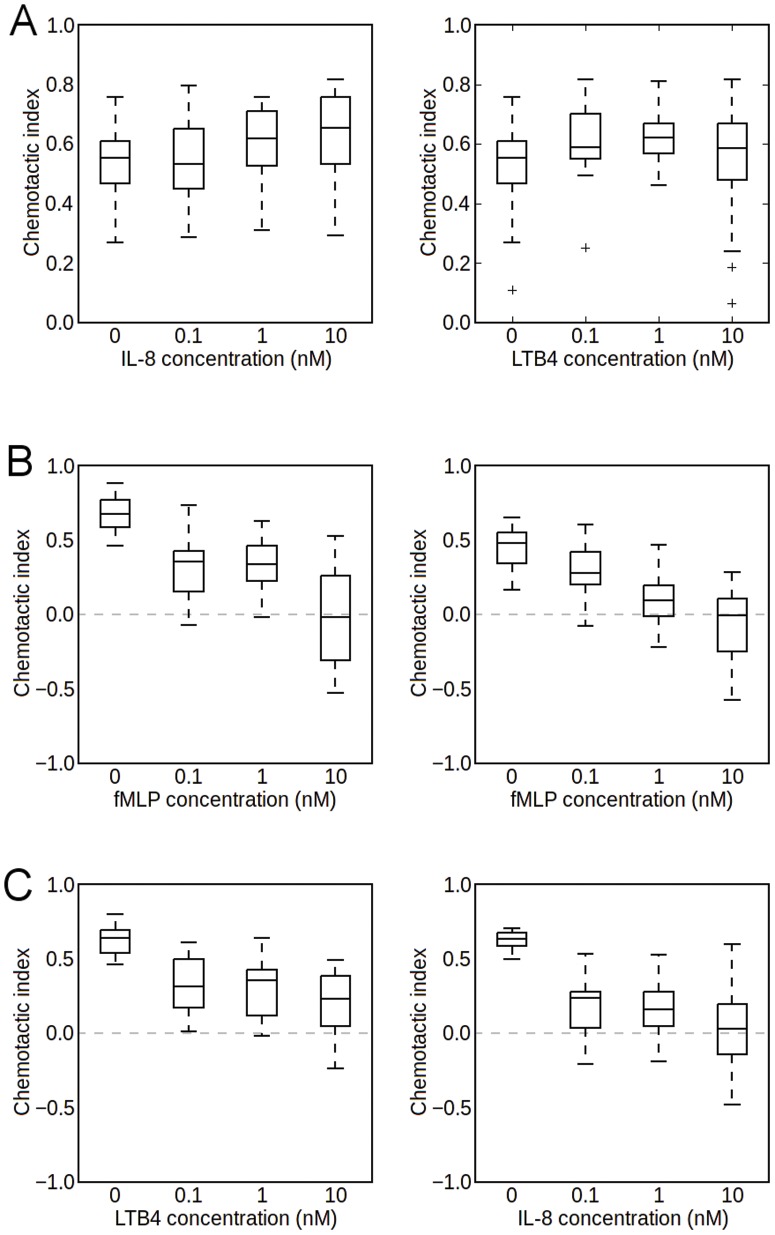
Chemotaxis in single gradients superimposed over isotropic attractant field. [A] Chemotactic index in a 0–25 nM fMLP gradient over uniform intermediate chemoattractant concentration. The fMLP gradient was fixed for all conditions, while the concentration of the uniform intermediate chemoattractant background was varied from 0 to 10 nM for both IL-8 and LTB_4_. 30 cells were tracked for 20 minutes for each experiment. [B] Chemotactic index in 0–25 nM IL-8 and 0–15 nM LTB_4_ gradients over uniform concentration of fMLP. All conditions in the left figure consisted of a fixed 0–25 nM IL-8 gradient with a uniform concentration of fMLP, while all conditions on the right had a fixed 0–15 nM LTB_4_ gradient with a uniform concentration of fMLP. 30 cells were tracked for each experiment. [C] Chemotactic indices for cells in intermediate chemoattractant gradients over uniform background concentration of alternate intermediate chemoattractant. All conditions in the left figure consisted of a fixed 0–15 nM IL-8 gradient over a varying uniform LTB_4_ background, while all conditions in the right figure consisted of a fixed 0–25 nM LTB_4_ gradient over a varying uniform IL-8 background.

To study the effect of crosstalk on chemotaxis, a single gradient of one chemoattractant was established over a uniform concentration of another. In the first case, an fMLP gradient was applied over uniform concentrations of IL-8 or LTB_4_, respectively. As shown in Figure 2A, we found that increasing the background chemoattractant concentrations does not inhibit chemotaxis up the fMLP gradient in either case. This is in agreement with previous findings [24], [25], [28], [31]. In the second case (Figure 2B), single gradients of the intermediate chemoattractants LTB_4_ and IL-8 were established over a uniform concentration of fMLP. We found that increasing the concentration of fMLP inhibited chemotaxis up either intermediate attractant gradient. Together with the previous result, this observation corroborates the existence of a signaling hierarchy between the two classes of chemoattractants, in which fMLP takes precedence over both IL-8 and LTB_4_ as previously described [31]. In the third case (Figure 2C), a single IL-8 gradient was established over a uniform concentration of LTB_4_, and vice versa. In this case, a negative correlation can be noted between the background concentration and chemotactic index, where the background chemoattractant appears to inhibit migration up the gradient of the other chemoattractant. These results suggest that neither intermediate chemoattractant takes precedence over the other in terms of a strong signaling hierarchy - instead, both species appear to attenuate the chemotactic efficiency toward the other in a relatively symmetric fashion.

### Oscillatory behavior in opposing intermediate attractant gradients

**Figure 3:**
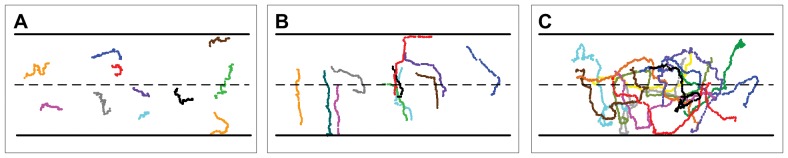
Sample cell trajectories under varying intermediate chemoattractant conditions. Representative cell trajectories indicating the migration behavior of cells in [A] isotropic conditions (50 nM LTB_4_), [B] single gradients (0–50 nM LTB_4_), and [C] dual opposing gradients of IL-8 (0–10 nM) and LTB_4_ (0–15 nM).

We next explored chemotaxis in opposing linear gradients of IL-8 and LTB_4_. In these experiments, the cells were tracked for up to 80 minutes to analyze their individual behavior. As shown in Figure 3C and Movies S3, the cells were found to migrate back and forth in the channel in an oscillatory manner. Representative trajectories of cells under varying gradient conditions are shown in Figures S3 and S4. The first thing to note is that in almost all cases, cells initially migrated down the more proximal gradient and up the more distant gradient. That is, cells initially positioned in the upper half of the channel appeared to move toward the lower half and vice-versa, as previously documented [25], [35]. Over longer times, however, we note that these cells then undergo multiple directional changes, resulting in oscillatory trajectories. While it was previously speculated that cells would move in this manner [35], this is the first experimental confirmation of this hypothesis.

**Figure 4:**
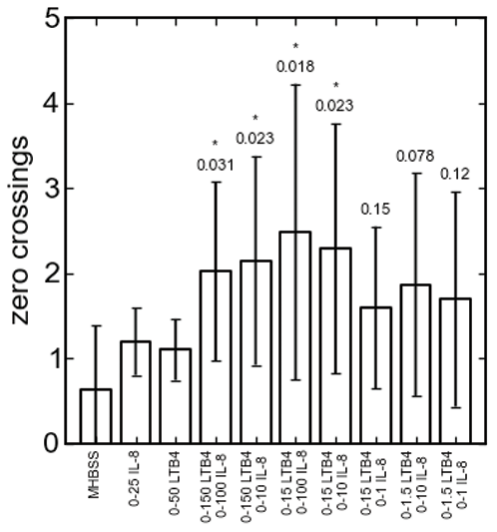
Oscillatory behavior based on zero crossings. The cell trajectories were analyzed to count the number of times the channel centerline was crossed within each 80 minute experiment. Noise was attenuated using state estimation via a standard Kalman filter with process noise variance set to 10^−4^ microns^2^. The first two columns on the left represent the single gradient controls for IL-8 and LTB_4_ respectively. P-values relate to single gradient controls, where * represents statistical significance compared to isotropic conditions (p<0.05). Statistical significance was determined using a one-tailed Welch's t-test of unequal variance on the data.

To demonstrate that the oscillations occur only when two gradients are present, we compared the trajectories in isotropic, single gradient, and dual gradient cases (Figure 3, Movies S1–S3). Unlike the trajectories for cells exposed to a uniform concentration of LTB_4_, in which displacement did not deviated far from the initial starting position, the trajectories in the dual gradient case oscillate around the middle of the channel. This difference is further shown quantitatively in Figure 4, where we counted the average number of times the channel median was crossed by each cell. Prior to counting, the data was first pre-processed using state estimation via a standard Kalman filter with process noise variance set to 10^−4^ microns^2^. Here, we see that the mean number of zero crossings is higher in the dual gradient experiments than in the control. Specifically, when both gradients are large (≥0–10 nM), the cells migrate across the center of the channel significantly more times than in either the single gradient or isotropic conditions. This result implies that neutrophils oscillate in opposing gradients of intermediate chemoattractants. The large variance occurs because of the random starting location of the cells. Still, this result suggests that the oscillatory behavior of cells in the opposing gradients is not the result of the random motion of migrating cells in isotropic conditions. To determine if neutrophils can preferentially migrate to end-target chemoattractants, opposing gradients of fMLP vs. IL-8 and fMLP vs. LTB_4_ were established in the microchannel. In this case, the cells migrate up the fMLP gradient (Figure S5) as seen previously [24], [25].

### Feedback-based model for neutrophil migration

To further understand oscillatory motion in opposing gradients of intermediate chemoattractants, we explored a number of different mathematical models of neutrophil chemotaxis. These models were phenomenological in the sense that they captured only the behavioral response of neutrophils and ignored the governing signal transduction pathways (details provided as Supporting Information). Analysis of these models indicates that sensory adaptation alone may be insufficient to generate oscillatory motion in linear gradients. In particular, no single choice of parameters would sustain oscillations over linear gradients of varying magnitude. Moreover, these models predict that the amplitude is dependent on the initial position of the cells contrary to what we observe experimentally. Finally, they all predict that the oscillations will decay exponentially, also contrary to what we observe albeit over the time course of our experiments. Therefore, we considered alternate mechanisms.

**Figure 5:**
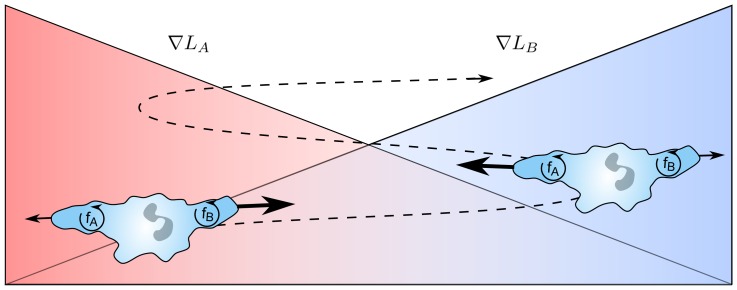
Schematic of proposed mechanism governing oscillatory motion. Oscillatory behavior results from the amplification of the response toward the distant chemokine source, while inhibition of the opposite signal results in the switch-like behavior. The variables 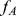 and 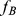 denote the positive feedback loops governing the switch behavior.

**Figure 6:**
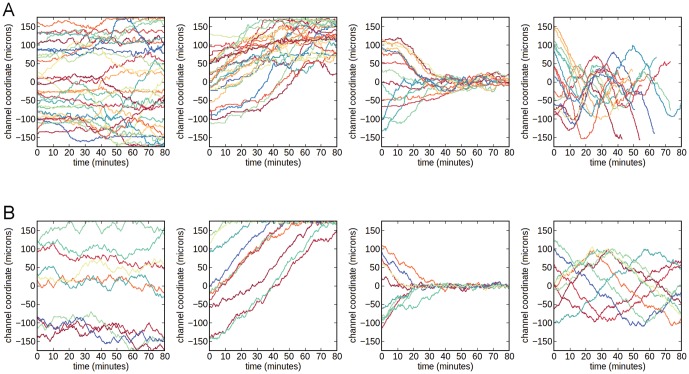
Comparison of theoretical and experimental neutrophil responses to varying conditions. [A] Cell trajectories along the channel width in our microfluidic experiments, under isotropic (25 nM IL-8), single linear gradient (0–25 nM IL-8), single hill-type gradient (0–25-0 nM IL-8), and dual opposing gradients of 150 nM LTB_4_ and 100 nM IL-8, respectively. The hill-type gradient was established using a three-inlet device with a similar design to the “Y-shaped” device. Confirmation of the concentration profile is shown in Figure S6. [B] Simulated cell positions along the channel width obtained from our feedback-based model, when applied to the corresponding chemoattractant conditions. The qualitative behaviors are similar. The parameter used for the simulation are: 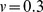 µm/s, 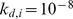 M, 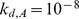 M, and 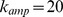.

One simple mechanism that would explain the oscillations is to assume that neutrophils selectively lock onto one chemoattractant while ignoring the other. Such a mechanism could arise if the signaling pathways employ positive feedback (Figure 5), as is known to occur in neutrophil chemotaxis [36], [37]. Assuming that these feedback loops are competitive, then the result would be a hysteretic switch that enables cells to lock onto specific chemoattractant gradients while ignoring others (details provided in the Materials and Methods section). Note, this mechanism can be viewed as a form of sensory adaptation, albeit in a competitive form. We tested this mechanism by extending a model previously developed by Van Haastert et al. [38]–[40] for chemotaxis towards a single chemoattractant. Application of this model to various experimental conditions, including (i) an isotropic chemoattractant environment, (ii) a single chemoattractant gradient, (iii) a single chemoattractant peak (or “hill gradient”), and (iv) dual opposing intermediary chemoattractant gradients, produces simulated cell trajectories that are qualitatively consistent with experimental results, as shown in Figure 6. While this mechanism is still speculative, it nonetheless supports the hypothesis that neutrophils make specific choices when given a menu of options.

## Discussion

Neutrophil chemotaxis is an important physiological process that occurs during immune defense and wound healing. During this process, neutrophils encounter chemoattractants emanating from multiple sources resulting in a complex milieu of conflicting chemoattractant gradients. More specifically, on the surface of the endothelium neutrophils are presented with opposing gradients of intermediate chemoattractants [21]–[23]. There, the cells must migrate away from the endothelial-derived attractant toward the source of other tissue-derived intermediate chemoattractants [41]. These cells must efficiently navigate through the chemoattractant landscape to reach the site of infection. However, the chemotactic response to multiple attractant sources remains poorly understood [1]–[3]. In this work, we applied a microfluidic device to study the behavior of primary cells under opposing gradients of the intermediate chemoattractants LTB_4_ and IL-8. Previous reports describe neutrophils seeking the distant source in opposing intermediate attractant gradients [25]. By increasing the length of the experiments, we found that neutrophils oscillate in opposing intermediate attractant gradients. In addition, the results corroborate previous reports of neutrophil responses in varying chemoattractant conditions [24], [25], showing that: (i) the intermediate chemoattractants IL-8 and LTB_4_ do not inhibit the response toward the end-target chemoattractant fMLP (indicating that the latter takes precedence in an intracellular signaling hierarchy), and that (ii) IL-8 and LTB_4_ have a weak inhibitory effect on one another, but their effect is mutual (suggesting that no hierarchy is present for these intermediate cues).

While many models of neutrophil chemotaxis have been proposed [38]–[40], [42]–[56], these models have largely focused on chemotaxis towards a single chemoattractant. Only a few models have explored network chemotaxis in presence of multiple chemoattractants. In one notable study, Lin and coworkers developed a mathematical model based on sensory adaptation [54], [57]. They showed that cells with desensitizable receptors could indeed exhibit preferential migration toward distant sources. However, this model does not predict oscillatory motion. Rather, their model predicts that the cells will migrate to a position equidistant between the two chemoattractant sources (in dimensionless terms). The reason this occurs is because the equidistant point is where both sets of receptors are equally desensitized. A key difference in our model is that only one set of receptors is being deactivated while the other remains active. Moreover, our model exhibits hysteresis which enables it to bypass the otherwise stable fixed point at the equidistant midpoint.

In a second notable study, Oelz and coworkers suggested that the migrational bias towards the distant chemoattractant was due to the cells' inability to rapidly adjust their sensitivities [55]. By allowing for dynamic sensitivities, their model predicts that cells can oscillate back and forth between the two sources under certain conditions. While this model predicts oscillatory movement, it does so for only exponential gradients and not linear ones (see Supporting Information). In addition, their model predicts that the amplitude of the oscillations is determined by the starting position of the cells. Our experimental results, on the other hand, suggest that the oscillatory response of cells is invariant to the phase or initial position of the cell in its trajectory. The robustness of the sustained oscillatory response strengthens the argument for a feedback-based mechanism in which cells transiently lock onto sensed targets. That said, their model provided the basis for our model.

The switch-like behavior, predicted by our model, could be a function of positive feedback mechanisms in intracellular signaling pathways. For instance, the lipid PtdIns(3,4,5)P(3) has been shown to stimulate its own accumulation by activating Rho GTPases, which in turn increase PtdIns(3,4,5)P(3) accumulation [36], [37]. This positive feedback mechanism allows signals to be amplified and the cell to polarize and respond in the direction of the strongest signal, allowing the cell to lock onto one chemoattractant gradient while ignoring others. A similar mechanism occurs during actin polymerization, where neutrophils responding to chemotactic stimuli increase the nucleation and polymerization of actin filaments in the region receiving maximal chemotactic stimulation [58]. In addition, the switching mechanism does not appear to be a phenomenon exclusive to chemotaxis in dual intermediate chemoattractant gradients. In the presence of both end target and intermediate chemoattractants, PTEN prioritizes these cues switching from the PI(3)K pathway towards a p38 MAPk pathway [26]. The process of switching between the two pathways allows the cells to prioritize and integrate responses to multiple chemotactic cues. In addition, recent discoveries have shown that *in vivo*, different chemoattractants may collaborate sequentially in temporal and spatial cascades to choreograph neutrophil recruitment [4], [59]. The requirement for particular chemoattractant types at specific steps in this process could involve unique temporal and/or spatial patterns of chemoattractant expression, but the corresponding sensory mechanism in migrating cells could be achieved through this switch-like response, in which multiple signals could be prioritized through internally designated response thresholds. In another recent study, neutrophil chemotaxis has been shown to be regulated by bidirectional regulation of distinct ‘stop’ and ‘go’ signals [60]. The cell can dynamically switch between the two signals to tightly regulate migration. Further, two-photon imaging of neutrophil chemotaxis in zebrafish showed that retrograde chemotaxis of cells away from the site of inflammation may also play an important role in inflammatory resolution [61]–[63]. The ability of cells to lock on and off of target cues may be central to this process, by allowing cells to move between different locations through tight regulation. Overall, these studies suggest a switch-like mechanism may help to regulate cell migration and find their end target.

**Figure 7:**
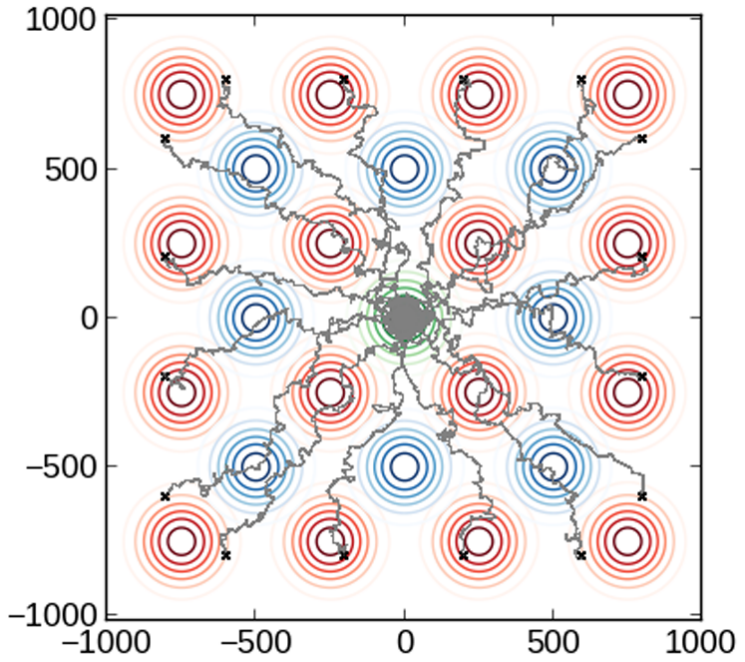
Simulation of stepwise navigation through multiple chemoattractant sources. Gray lines denote the trajectories of individual cells. Red and blue lines denote the contours for Gaussian concentration profiles for intermediate chemoattractant sources and the green lines denote the contours for an end-target chemoattractant source (positioned in the center of the plot). Note the cells migrate between intermediate sources of chemoattractants before converging on the final end target. The parameter values used in this simulation are: 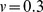 µm/s, 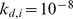 M, 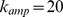, and 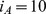. All chemoattractant sources were modeled as Gaussians: 
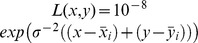
where 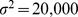 µm^2^ and 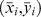
denote the position of the source.

In the context of physiological environments, particularly in the extravascular space, the consequences of a switch-like chemotactic response and migration toward distant intermediate attractant sources remain unclear. One hypothesis, in line with that proposed by Foxman and coworkers [35], is that this response might enable the cells to navigate long distances in a stepwise fashion between a network of intermediate chemoattractant sources, as a way to increase their chances of locating end targets. In support of this hypothesis, recent discoveries by Lammermann and coworkers have shown that *in vivo* LTB_4_ acts as an intercellular signal relay molecule, where LTB_4_ amplifies local cell death signals and enhances the radius of highly directed neutrophil migration [64]. Furthermore, defects in the generation of these intermediate chemoattractant gradients leads to ill-favored accumulation of neutrophils in tissue [4], [59]. This guided homing mechanism may work to enhance the search efficiency of neutrophils when multiple stimuli are present, by using the sequential intermediate attractant sources for loose guidance *en route* to their destinations, as shown in Figure 7.

## Materials and Methods

### Microfluidic device fabrication

The microfluidic device was comprised of a molded poly(dimethylsiloxane) (PDMS, General Electric RTV 650 Part A/B) slab bonded to a glass substrate. High-resolution printing (5080 dpi) was used to print the mask with the design pattern on a transparency film. The mask was used to fabricate 50 µm high SU-8 2050 photoresist (Microchem) features on a silicon wafer via photolithography. PDMS molds with embossed channels were fabricated using soft lithography by curing the pre-polymer on the silicon master for 2 hours at 70°C. The PDMS replica was then peeled off the silicon master. Inlets and outlets for the fluids and cells were created in PDMS using a steel punch. The surface of the PDMS replica and a clean glass coverslip (Fisher Scientific) were treated with air plasma for 90 seconds (Model PDC-001, Harrick Scientific) and irreversibly bonded to complete the device assembly (Figure S7). The device inlets were then connected to 1 mL syringes (BD Biosciences) with 23 G ¾ size needles (BD Biosciences) via PTFE tubing (Cole-Parmer). All syringes were calibrated and pushed by a constant pressure syringe pump (Harvard Apparatus). Prior to each experiment, the device was also loaded with fibronectin (25 µg/mL, Invitrogen) and kept at room temperature for 30 minutes to promote optimal cell adhesion.

### Gradient formation

The concentration gradients across the microchannel were verified by infusing fluorescently-labeled solution (Fluorescein, Sigma Aldrich) from one inlet and an unlabeled solution from the other inlet of the device (Figure S1). Diffusive mixing across the interface of the laminar streams led to formation of the gradient. Fluorescent images were acquired at different locations along the channel using a FITC filter on the Zeiss Axiovert 200M microscope. ImageJ was then used to analyze the fluorescence intensity profiles. The plotted profiles confirm the formation of a well-defined, linear and stable concentration gradient as also reported in similar works [65].

### Primary neutrophil isolation

Sodium heparin (Fisher Scientific) anti-coagulated human blood was obtained from healthy volunteers according to approved University of Illinois at Urbana-Champaign Institutional Review Board (IRB) protocol 12030. Written consent was obtained from all participants prior to any blood draw according to the consent procedure approved by the IRB. Neutrophils were isolated by density gradient centrifugation of a centrifuge tube containing 4 mL of whole blood layered over 4 mL of Cell Isolation Medium (Cedar Lane Labs). The isolated neutrophils were washed twice and resuspended to 10^7^ cells/mL in Hank's Balanced Salt Solution with 2% human serum albumin (HSA) and incubated at 37°C following a previously reported protocol [66].

### Cell preparation

Cells were washed and suspended in modified Hank's Balanced Salt Solution (mHBSS) containing 1% HSA. The device was prepared by washing the channels with a 70% v/v ethanol solution. The channels were then rinsed with phosphate buffered saline and 30 µL of the neutrophil suspension (5×10^6^ cells/mL) was injected into the microfluidic device. The device was next incubated for 20 minutes to allow cells to adhere to the substrate. After incubation, the device was connected to a syringe pump and the desired combination of chemoattractant solutions (IL-8 and fMLP Sigma Aldrich, LTB_4_ Fisher Scientific) were infused into the device from separate inlets at a flow rate of 0.02 mL/hr to establish the desired concentration gradients.

### Time-lapse microscopy and analysis

Upon visual confirmation of a stable gradient, differential interference contrast (DIC) images were captured with a Zeiss 10X NA 1.30 DIC objective on a Zeiss Axiovert 200 M microscope every 10 seconds. All images were captured with a cooled charge-coupled device camera (AxioCam MR3, Zeiss). Cells were then randomly selected from the image stack and manually tracked using ImageJ® (NIH) using the Manual Tracking plugin by Fabrice Cordelieres (Institut Curie, France). The plugin provided a way to tabulate the XY coordinates of each cell centroid in the temporal stack, as well as to obtain velocity and displacement measurements between successive frames. The resulting excel spreadsheets were then analyzed using custom Python scripts to yield cell trajectories, chemotactic indices, cell speeds and mean square displacements. We define the chemotactic index (CI) as displacement along the gradient direction (x) over the total migration distance (d), or CI = x/d, while the mean square displacement is defined as: MSD = 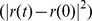.

### Detailed description of neutrophil model

Our model is based on a pseudopod-based model previously developed by van Haastert [38], [52]. Cell motion is modeled as a correlated random walk (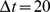 seconds), where each time step corresponds to the next pseudopod generated. Following van Haastert, we assume that the next pseudopod is generated either by splitting off from an existing one in a direction biased by the external chemoattractant gradients or by de novo pseudopod formation, where the new pseudopod is generated in an entirely random direction (see below for further details). Where this model differs from previous ones is that we account for multiple chemoattractants and include an autocatalytic feedback loop that enables the cell to lock onto one chemoattractant while ignore the other.

**Figure 8:**
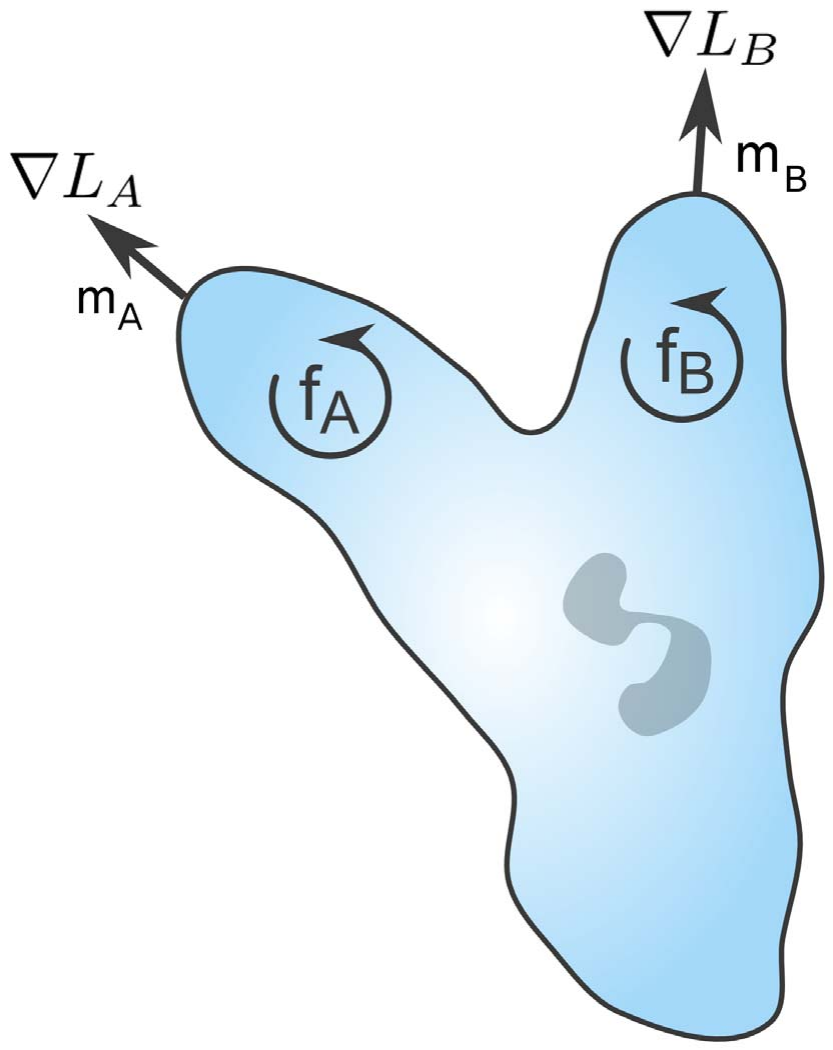
Schematic of proposed mechanism for signal discrimination. The response toward each chemoattractant is autocatalyzed in a nonlinear fashion, where 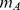 and 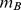 denote the normalized response to each attractant.

In the case of two intermediate chemoattractants, denoted by A and B, we assume the target direction of the cell 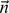 is determined by the weighted sum of the associated chemoattractant gradients:
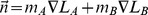
where 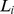 denotes the concentration for chemoattractant i and 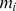 the associated weights (**Figure 8**). The values for the weights are determined by the following set of differential equations
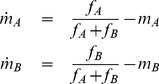
where 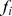 denotes the amplified response to chemoattractant i and is given by the expression
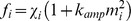
where 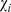 is chemotaxis coefficient for chemoattractant i and the 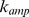 is the amplification gain. This functional form was chosen so that 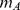 and 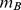 sum to one and to provide a simple mechanism for hysteresis. As shown in Figure S8, 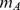 dominates (i.e. 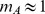) when 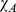 is larger than 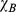, and vice versa. In other words, this mechanism enables cells to lock onto one chemoattractant signal while ignoring the other. Such a mechanism, we believe, provides the simplest explanation for the observed oscillatory motion in competing gradients of chemoattractants. While there are a number of mechanisms that could give rise to this behavior, including the excitable networks previously proposed for eukaryotic chemotaxis [42], [47], we chose to focus on a simple phenomenological model known to exhibit switch-like behavior as the molecular details governing chemotaxis to multiple gradients are still unknown.

The chemotaxis coefficient 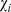 determines how strongly the cells are biased in the direction 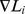. We assumed that the chemotaxis coefficient is given by the expression
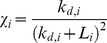
where the 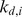 denotes the dissociation constant for the chemoattractant i. Note that if the fraction of bound receptors for chemoattractant i is given by
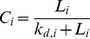
then
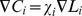



In other words, the cells move in the direction where the number of bound receptors on their surface is greatest. Implicit in this formulation is the assumption that each chemoattractant binds a distinct receptor type and that there is no crosstalk between these receptors.

The directional bias 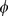 of splitting pseudopods is given by the expression
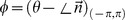
where 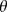 denotes the current direction of the pseudopod. The difference is taken to be the minimal distance on the periodic domain 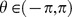. This aspect of the model coincides with compass-based theories in which an intracellular compass dictates the subsequent direction of motion.

Following van Haastert, we simulate cell motion by assuming that the direction of the newly split pseudopod is given by
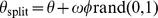
where 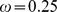 denotes the extent to which a pseudopod can split in a given step. Again, following the Van Haastert model, we also incorporate additional randomness by allowing variability in the new pseudopod direction. In our model, we sample 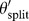 from a von Mises distribution whose mean is the new target direction 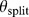 and variance is 20°. The position of the cell is then updated using the following set of equations
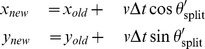



We also assumed that de novo pseudopods are formed with a probability 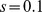 at each time step. If such occurs, the new direction of the cell is given by
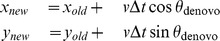
where 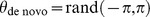.

We can readily extend the model to account for chemotaxis in opposing gradients of end-target chemoattractants such as fMLP and intermediate chemoattractants such as IL-8 and LTB4 by modifying the sensitivity equations as follows
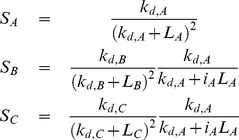
where the subscripts A, B, and C denote fMLP, IL-8, and LTB4, respectively. The parameter 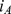 is the strength of inhibition and set equal to 10 in our simulations. Note that 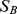 and 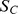 are inhibited when the concentration of the end-target chemoattractant, denoted by A, is high. The remaining equations are similar to as before:
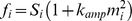
and
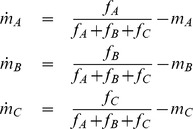



The response vector is given by
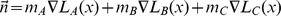



Otherwise, the model is identical. As can be seen in Figure S9, this extension enables us to recapitulate the experiments involving opposing gradients of fMLP and IL-8 or LTB4.

The primary justification for this model is that it supports our experimental observation. In particular, the model captures the oscillatory motion of the cell in opposing gradients of chemoattractants. While the true mechanism may be different than the one proposed here, our simple model still allows cells to sequentially lock onto targets as they migrate within opposing linear gradients. In the absence of such a mechanism, cells are predicted to migrate to a position equidistant between the two sources (Figure S10). Our model is in close agreement with existing models in the literature, albeit it at the phenomenological level, which suggest that an excitable system could explain many aspects of cell behavior, including spontaneous polarization, adaptation, and the high degree of signal amplification seen in cells [67], [68]. In our particular example with two stimuli, this type of thresholding mechanism gives rise to an ultrasensitive, “winner-take-all” type switch that is robust and yields similar amplitude irrespective of initial position, as observed experimentally (Figure S11).

## Supporting Information

Figure S1 **Cross-sectional concentration profile for single gradient.** Gradient formation was verified by feeding a fluorescein-labeled solution into one inlet and an unlabeled solution into the other inlet of the device. The resulting fluorescence intensity profile confirms the formation of a well-defined, stable, linear concentration gradient. The normalized FITC concentration across the channel cross-section is shown.
(TIF)

Figure S2 **Migration in isotropic attractant conditions.** [A] Uniform chemoattractant environments were established by flowing the same solution into both channel inlets. Cells were tracked for 20 minutes in fMLP, IL-8 and LTB4, and the upward migration indices of 30 cells are shown here for comparison against the control (MHBSS buffer only). [B] The mean square displacements (MSD) of the cells from the previous figure as a function of time. Cells were exposed to uniform concentrations of fMLP, IL-8 and LTB4. [C] The average linear velocities of the cells from the previous figure. Again, cells were exposed to uniform concentrations of fMLP, IL-8 and LTB4. [D] Sample trajectories from the previous control experiments. [top left] MHBSS buffer only; [top right] 25 nM fMLP; [bottom left] 25 nM IL-8; [bottom right] 50 nM LTB4.
(TIF)

Figure S3 **Sample cell trajectories in dual opposing intermediate chemoattractant gradients.** Representative cell trajectories indicating the migration behavior of cells in dual opposing gradients of IL-8 and LTB_4_.
(TIF)

Figure S4 **Aligned sample trajectories in dual opposing intermediate chemoattractant gradients.** As a visual guide, the cell trajectories from Figure S3 were aligned based on the farthest each cell migrated towards the IL-8 source (denoted zero time).
(TIF)

Figure S5 **Chemotaxis in opposing linear chemoattractant gradients with fMLP.** Chemotactic index in a 0–25 nM fMLP gradient versus varying IL-8 and LTB4 gradients. The fMLP gradient was fixed for all conditions, while the intermediate attractant gradient was varied from no gradient to 0–100 nM for both IL-8 and LTB4. 30 cells were tracked for 40 minutes for each experiment. The correlation with the intermediate chemoattractant gradient was weak with Pearson correlations (r. −0.1694; P. 0.1331) and (r. −0.1304; P. 0.1785), respectively.
(TIF)

Figure S6 **Cross-sectional concentration profile for hill-type gradient.** Gradient formation was verified by feeding a fluorescein-labeled solution into the central inlet and an unlabeled solution into the outer inlets. The normalized FITC concentration across the channel cross-section is shown.
(TIF)

Figure S7 **Schematic of microfluidic platform with Y-shaped channel.** The platform was comprised of a molded PDMS slab embossed with microchannels and bonded to a glass coverslip.
(TIF)

Figure S8
**The signaling threshold mechanism.** [Left] Response as function of 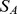 and 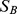 (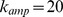). Note the hysteresis in the response – this is necessary to generate the oscillations that results from the overshoot inherent in this mechanism. [Right] Response as a function of amplification gain parameter 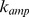 when 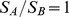.(TIF)

Figure S9
**Simulation of the model in a competing gradient of an end-target chemoattractant and intermediate chemoattractant.** The parameter values used in this simulation are: 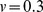 µm/s, 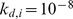 M, 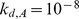 M, 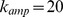, 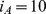, 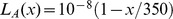 M (end-target), and 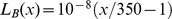 M (intermediate).(TIF)

Figure S10
**No oscillations are observed in the absence of pseudopod memory.** In these simulations, 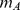 and 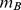 are fixed at the value 1.0. The parameter values used in this simulation are: 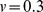 µm/s, 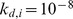 M, 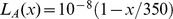
M, and 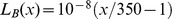 M.(TIF)

Figure S11
**The model is able to robustly generate sustained oscillatory behavior.** The initial position of the cells does not affect the amplitude of the oscillatory motion. The parameter values used in this simulation are: 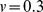 µm/s, 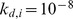 M, 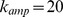,
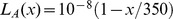
M, and 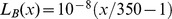 M.(TIF)

Text S1 **Analysis of alternate chemotaxis models.**
(PDF)

Movie S1 **Movie of neutrophil migration in an isotropic environment.** Neutrophil migration in a uniform chemoattractant environment of 50 nM LTB_4_. Cells were tracked to ease visual confirmation of migration patterns.
(AVI)

Movie S2 **Movie of neutrophil migration in a single attractant gradient.** Neutrophil chemotaxis in a single gradient of 0–50 nM LTB_4_. Cells were tracked to ease visual confirmation of migration patterns.
(AVI)

Movie S3 **Movie of neutrophil migration in dual opposing intermediary attractant gradients.** Neutrophil chemotaxis in dual opposing chemoattractant gradients of 10 nM IL-8 and 15 nM LTB_4_. Cells were tracked to ease visual confirmation of migration patterns.
(AVI)

## References

[1] Kono H, Rock KL (2008) How dying cells alert the immune system to danger. Nat Rev Immunol 8: 279–289. 18340345 10.1038/nri2215 PMC2763408

[2] Mantovani A, Cassatella MA, Costantini C, Jaillon S (2011) Neutrophils in the activation and regulation of innate and adaptive immunity. Nat Rev Immunol 11: 519–531. 21785456 10.1038/nri3024

[3] Luster AD, Alon R, von Andrian UH (2005) Immune cell migration in inflammation: present and future therapeutic targets. Nat Immunol 6: 1182–1190. 16369557 10.1038/ni1275

[4] McDonald B, Pittman K, Menezes GB, Hirota SA, Slaba I, et al (2010) Intravascular danger signals guide neutrophils to sites of sterile inflammation. Science 330: 362–366. 20947763 10.1126/science.1195491

[5] Afonso PV, Janka-Junttila M, Lee YJ, McCann CP, Oliver CM, et al (2012) LTB4 is a signal-relay molecule during neutrophil chemotaxis. Dev Cell 22: 1079–1091. 22542839 10.1016/j.devcel.2012.02.003 PMC4141281

[6] Cavicchioni G, Fraulini A, Falzarano S, Spisani S (2009) Oligomeric formylpeptide activity on human neutrophils. Eur J Med Chem 44: 4926–4930. 19748709 10.1016/j.ejmech.2009.08.010

[7] Servant G, Weiner OD, Herzmark P, Balla T, Sedat JW, et al (2000) Polarization of chemoattractant receptor signaling during neutrophil chemotaxis. Science 287: 1037–1040. 10669415 10.1126/science.287.5455.1037 PMC2822871

[8] Heit B, Liu L, Colarusso P, Puri KD, Kubes P (2008) PI3K accelerates, but is not required for, neutrophil chemotaxis to fMLP. J Cell Sci 121: 205–214. 18187452 10.1242/jcs.020412

[9] Adams DH, Lloyd AR (1997) Chemokines: leucocyte recruitment and activation cytokines. Lancet 349: 490–495. 9040590 10.1016/s0140-6736(96)07524-1

[10] Bleul CC, Fuhlbrigge RC, Casasnovas JM, Aiuti A, Springer TA (1996) A highly efficacious lymphocyte chemoattractant, stromal cell-derived factor 1 (SDF-1). J Exp Med 184: 1101–1109. 9064327 10.1084/jem.184.3.1101 PMC2192798

[11] Barnett ML, Lamb KA, Costello KM, Pike MC (1993) Characterization of interleukin-8 receptors in human neutrophil membranes: regulation by guanine nucleotides. Biochim Biophys Acta 1177: 275–282. 8323978 10.1016/0167-4889(93)90123-7

[12] Kreisle RA, Parker CW (1983) Specific binding of leukotriene B4 to a receptor on human polymorphonuclear leukocytes. J Exp Med 157: 628–641. 6296265 10.1084/jem.157.2.628 PMC2186934

[13] Li Jeon N, Baskaran H, Dertinger SK, Whitesides GM, Van de Water L, et al (2002) Neutrophil chemotaxis in linear and complex gradients of interleukin-8 formed in a microfabricated device. Nat Biotechnol 20: 826–830. 12091913 10.1038/nbt712

[14] Mathis SP, Jala VR, Lee DM, Haribabu B (2010) Nonredundant roles for leukotriene B4 receptors BLT1 and BLT2 in inflammatory arthritis. J Immunol 185: 3049–3056. 20656922 10.4049/jimmunol.1001031

[15] Reilly IA, Knapp HR, Fitzgerald GA (1988) Leukotriene B4 synthesis and neutrophil chemotaxis in chronic granulocytic leukaemia. J Clin Pathol 41: 1163–1167. 2850300 10.1136/jcp.41.11.1163 PMC1141723

[16] Sager R, Haskill S, Anisowicz A, Trask D, Pike MC (1991) GRO: a novel chemotactic cytokine. Adv Exp Med Biol 305: 73–77. 1755381 10.1007/978-1-4684-6009-4_9

[17] Gallin JI, Wright DG, Schiffmann E (1978) Role of secretory events in modulating human neutrophil chemotaxis. J Clin Invest 62: 1364–1374. 372235 10.1172/JCI109257 PMC371902

[18] Fernandez HN, Henson PM, Otani A, Hugli TE (1978) Chemotactic response to human C3a and C5a anaphylatoxins. I. Evaluation of C3a and C5a leukotaxis in vitro and under stimulated in vivo conditions. J Immunol 120: 109–115. 342601

[19] Niethammer P, Grabher C, Look AT, Mitchison TJ (2009) A tissue-scale gradient of hydrogen peroxide mediates rapid wound detection in zebrafish. Nature 459: 996–999. 19494811 10.1038/nature08119 PMC2803098

[20] Lewis MS, Whatley RE, Cain P, McIntyre TM, Prescott SM, et al (1988) Hydrogen peroxide stimulates the synthesis of platelet-activating factor by endothelium and induces endothelial cell-dependent neutrophil adhesion. J Clin Invest 82: 2045–2055. 3198764 10.1172/JCI113825 PMC442787

[21] Ley K, Laudanna C, Cybulsky MI, Nourshargh S (2007) Getting to the site of inflammation: the leukocyte adhesion cascade updated. Nat Rev Immunol 7: 678–689. 17717539 10.1038/nri2156

[22] Johnson Z, Proudfoot AE, Handel TM (2005) Interaction of chemokines and glycosaminoglycans: a new twist in the regulation of chemokine function with opportunities for therapeutic intervention. Cytokine Growth Factor Rev 16: 625–636. 15990353 10.1016/j.cytogfr.2005.04.006

[23] Middleton J, Neil S, Wintle J, Clark-Lewis I, Moore H, et al (1997) Transcytosis and surface presentation of IL-8 by venular endothelial cells. Cell 91: 385–395. 9363947 10.1016/s0092-8674(00)80422-5

[24] Heit B, Tavener S, Raharjo E, Kubes P (2002) An intracellular signaling hierarchy determines direction of migration in opposing chemotactic gradients. J Cell Biol 159: 91–102. 12370241 10.1083/jcb.200202114 PMC2173486

[25] Foxman EF, Campbell JJ, Butcher EC (1997) Multistep navigation and the combinatorial control of leukocyte chemotaxis. J Cell Biol 139: 1349–1360. 9382879 10.1083/jcb.139.5.1349 PMC2140208

[26] Heit B, Robbins SM, Downey CM, Guan Z, Colarusso P, et al (2008) PTEN functions to ‘prioritize’ chemotactic cues and prevent ‘distraction’ in migrating neutrophils. Nat Immunol 9: 743–752. 18536720 10.1038/ni.1623

[27] Lin F, Nguyen CM, Wang SJ, Saadi W, Gross SP, et al (2005) Neutrophil migration in opposing chemoattractant gradients using microfluidic chemotaxis devices. Ann Biomed Eng 33: 475–482. 15909653 10.1007/s10439-005-2503-6

[28] Kim DH, Christy L (2012) Neutrophil Chemotaxis within a Competing Gradient of Chemoattractants. Analytical Chemistry. 10.1021/ac3009548 PMC3404751 22816782

[29] Li Z, Dong X, Wang Z, Liu W, Deng N, et al (2005) Regulation of PTEN by Rho small GTPases. Nat Cell Biol 7: 399–404. 15793569 10.1038/ncb1236

[30] Billadeau DD (2008) PTEN gives neutrophils direction. Nat Immunol 9: 716–718. 18563079 10.1038/ni0708-716

[31] Campbell JJ, Foxman EF, Butcher EC (1997) Chemoattractant receptor cross talk as a regulatory mechanism in leukocyte adhesion and migration. Eur J Immunol 27: 2571–2578. 9368612 10.1002/eji.1830271016

[32] Lin F, Nguyen CM, Wang SJ, Saadi W, Gross SP, et al (2004) Effective neutrophil chemotaxis is strongly influenced by mean IL-8 concentration. Biochem Biophys Res Commun 319: 576–581. 15178445 10.1016/j.bbrc.2004.05.029

[33] Allan RB, Wilkinson PC (1978) A visual analysis of chemotactic and chemokinetic locomotion of human neutrophil leucocytes. Use of a new chemotaxis assay with Candida albicans as gradient source. Exp Cell Res 111: 191–203. 340239 10.1016/0014-4827(78)90249-5

[34] Tharp WG, Yadav R, Irimia D, Upadhyaya A, Samadani A, et al (2006) Neutrophil chemorepulsion in defined interleukin-8 gradients in vitro and in vivo. J Leukoc Biol 79: 539–554. 16365152 10.1189/jlb.0905516

[35] Foxman EF, Kunkel EJ, Butcher EC (1999) Integrating conflicting chemotactic signals. The role of memory in leukocyte navigation. J Cell Biol 147: 577–588. 10545501 10.1083/jcb.147.3.577 PMC2151176

[36] Wang F, Herzmark P, Weiner OD, Srinivasan S, Servant G, et al (2002) Lipid products of PI(3)Ks maintain persistent cell polarity and directed motility in neutrophils. Nat Cell Biol 4: 513–518. 12080345 10.1038/ncb810

[37] Weiner OD, Neilsen PO, Prestwich GD, Kirschner MW, Cantley LC, et al (2002) A PtdInsP(3)- and Rho GTPase-mediated positive feedback loop regulates neutrophil polarity. Nat Cell Biol 4: 509–513. 12080346 10.1038/ncb811 PMC2823287

[38] Bosgraaf L, Van Haastert PJ (2009) Navigation of chemotactic cells by parallel signaling to pseudopod persistence and orientation. PLoS One 4: e6842. 19718261 10.1371/journal.pone.0006842 PMC2729408

[39] Bosgraaf L, Van Haastert PJ (2009) The ordered extension of pseudopodia by amoeboid cells in the absence of external cues. PLoS One 4: e5253. 19384419 10.1371/journal.pone.0005253 PMC2668753

[40] Neilson MP, Veltman DM, van Haastert PJ, Webb SD, Mackenzie JA, et al (2011) Chemotaxis: a feedback-based computational model robustly predicts multiple aspects of real cell behaviour. PLoS Biol 9: e1000618. 21610858 10.1371/journal.pbio.1000618 PMC3096608

[41] Dimasi D, Sun WY, Bonder CS (2013) Neutrophil interactions with the vascular endothelium. Int Immunopharmacol. 10.1016/j.intimp.2013.05.034 23863858

[42] Xiong Y, Huang CH, Iglesias PA, Devreotes PN (2010) Cells navigate with a local-excitation, global-inhibition-biased excitable network. Proc Natl Acad Sci U S A 107: 17079–17086. 20864631 10.1073/pnas.1011271107 PMC2951443

[43] Levine H, Kessler DA, Rappel WJ (2006) Directional sensing in eukaryotic chemotaxis: a balanced inactivation model. Proc Natl Acad Sci U S A 103: 9761–9766. 16782813 10.1073/pnas.0601302103 PMC1502527

[44] Onsum M, Rao CV (2007) A mathematical model for neutrophil gradient sensing and polarization. PLoS Comput Biol 3: e36. 17367201 10.1371/journal.pcbi.0030036 PMC1828701

[45] Insall RH (2010) Understanding eukaryotic chemotaxis: a pseudopod-centred view. Nat Rev Mol Cell Biol 11: 453–458. 20445546 10.1038/nrm2905

[46] Andrew N, Insall RH (2007) Chemotaxis in shallow gradients is mediated independently of PtdIns 3-kinase by biased choices between random protrusions. Nat Cell Biol 9: 193–200. 17220879 10.1038/ncb1536

[47] Ma L, Janetopoulos C, Yang L, Devreotes PN, Iglesias PA (2004) Two complementary, local excitation, global inhibition mechanisms acting in parallel can explain the chemoattractant-induced regulation of PI(3,4,5)P3 response in dictyostelium cells. Biophys J 87: 3764–3774. 15465874 10.1529/biophysj.104.045484 PMC1304889

[48] Beta C, Amselem G, Bodenschatz E (2008) A bistable mechanism for directional sensing. New Journal of Physics 10..

[49] Maree AF, Jilkine A, Dawes A, Grieneisen VA, Edelstein-Keshet L (2006) Polarization and movement of keratocytes: a multiscale modelling approach. Bull Math Biol 68: 1169–1211. 16794915 10.1007/s11538-006-9131-7

[50] Brandman O, Ferrell JE Jr, Li R, Meyer T (2005) Interlinked fast and slow positive feedback loops drive reliable cell decisions. Science 310: 496–498. 16239477 10.1126/science.1113834 PMC3175767

[51] Millius A, Dandekar SN, Houk AR, Weiner OD (2009) Neutrophils establish rapid and robust WAVE complex polarity in an actin-dependent fashion. Curr Biol 19: 253–259. 19200726 10.1016/j.cub.2008.12.044 PMC2705202

[52] Van Haastert PJ (2010) A model for a correlated random walk based on the ordered extension of pseudopodia. PLoS Comput Biol 6.. 10.1371/journal.pcbi.1000874 PMC2920832 20711349

[53] Van Haastert PJ (2010) A stochastic model for chemotaxis based on the ordered extension of pseudopods. Biophys J 99: 3345–3354. 21081083 10.1016/j.bpj.2010.09.042 PMC2980707

[54] Wu D, Lin F (2011) Modeling cell gradient sensing and migration in competing chemoattractant fields. PLoS One 6: e18805. 21559528 10.1371/journal.pone.0018805 PMC3084714

[55] Oelz D, Schmeiser C, Soreff A (2005) Multistep navigation of leukocytes: a stochastic model with memory effects. Math Med Biol 22: 291–303. 16203749 10.1093/imammb/dqi009

[56] Jilkine A, Edelstein-Keshet L (2011) A comparison of mathematical models for polarization of single eukaryotic cells in response to guided cues. PLoS Comput Biol 7: e1001121. 21552548 10.1371/journal.pcbi.1001121 PMC3084230

[57] Lin F, Butcher EC (2008) Modeling the role of homologous receptor desensitization in cell gradient sensing. J Immunol 181: 8335–8343. 19050250 10.4049/jimmunol.181.12.8335 PMC2596625

[58] Weiner OD, Servant G, Welch MD, Mitchison TJ, Sedat JW, et al (1999) Spatial control of actin polymerization during neutrophil chemotaxis. Nat Cell Biol 1: 75–81. 10559877 10.1038/10042 PMC2828058

[59] Chou RC, Kim ND, Sadik CD, Seung E, Lan Y, et al (2010) Lipid-cytokine-chemokine cascade drives neutrophil recruitment in a murine model of inflammatory arthritis. Immunity 33: 266–278. 20727790 10.1016/j.immuni.2010.07.018 PMC3155777

[60] Liu X, Ma B, Malik AB, Tang H, Yang T, et al (2012) Bidirectional regulation of neutrophil migration by mitogen-activated protein kinases. Nat Immunol 13: 457–464. 22447027 10.1038/ni.2258 PMC3330201

[61] Mathias JR, Perrin BJ, Liu TX, Kanki J, Look AT, et al (2006) Resolution of inflammation by retrograde chemotaxis of neutrophils in transgenic zebrafish. J Leukoc Biol 80: 1281–1288. 16963624 10.1189/jlb.0506346

[62] Mathias JR, Walters KB, Huttenlocher A (2009) Neutrophil motility in vivo using zebrafish. Methods Mol Biol 571: 151–166. 19763965 10.1007/978-1-60761-198-1_10

[63] Benard EL, van der Sar AM, Ellett F, Lieschke GJ, Spaink HP, et al.. (2012) Infection of zebrafish embryos with intracellular bacterial pathogens. J Vis Exp. 10.3791/3781 PMC3415172 22453760

[64] Lammermann T, Afonso PV, Angermann BR, Wang JM, Kastenmuller W, et al (2013) Neutrophil swarms require LTB4 and integrins at sites of cell death in vivo. Nature 498: 371–375. 23708969 10.1038/nature12175 PMC3879961

[65] Lin F, Butcher EC (2006) T cell chemotaxis in a simple microfluidic device. Lab Chip 6: 1462–1469. 17066171 10.1039/b607071j

[66] Oh H, Siano B, Diamond S (2008) Neutrophil isolation protocol. J Vis Exp. 10.3791/745 PMC3074468 19066523

[67] Iglesias PA, Devreotes PN (2012) Biased excitable networks: how cells direct motion in response to gradients. Curr Opin Cell Biol 24: 245–253. 22154943 10.1016/j.ceb.2011.11.009 PMC3415256

[68] Porter JR, Andrews BW, Iglesias PA (2012) A framework for designing and analyzing binary decision-making strategies in cellular systems. Integr Biol (Camb) 4: 310–317. 22370552 10.1039/C2IB90009B PMC4547352

