# Supplementary figures and images for: Oscillatory Behavior of Neutrophils under Opposing Chemoattractant Gradients Supports a Winner-Take-All Mechanism
Source: PLoS One. 2014 Jan 21;9(1):e85726. doi: 10.1371/journal.pone.0085726 (PMC3897492; doi:10.1371/journal.pone.0085726)

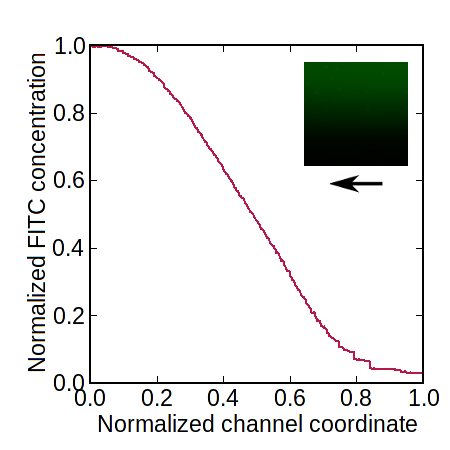

Supplement: Figure S1 — Cross-sectional concentration profile for single gradient. Gradient formation was verified by feeding a fluorescein-labeled solution into one inlet and an unlabeled solution into the other inlet of the device. The resulting fluorescence intensity profile confirms the formation of a well-defined, stable, linear concentration gradient. The normalized FITC concentration across the channel cross-section is shown. (TIF) [file pone.0085726.s001.tif]

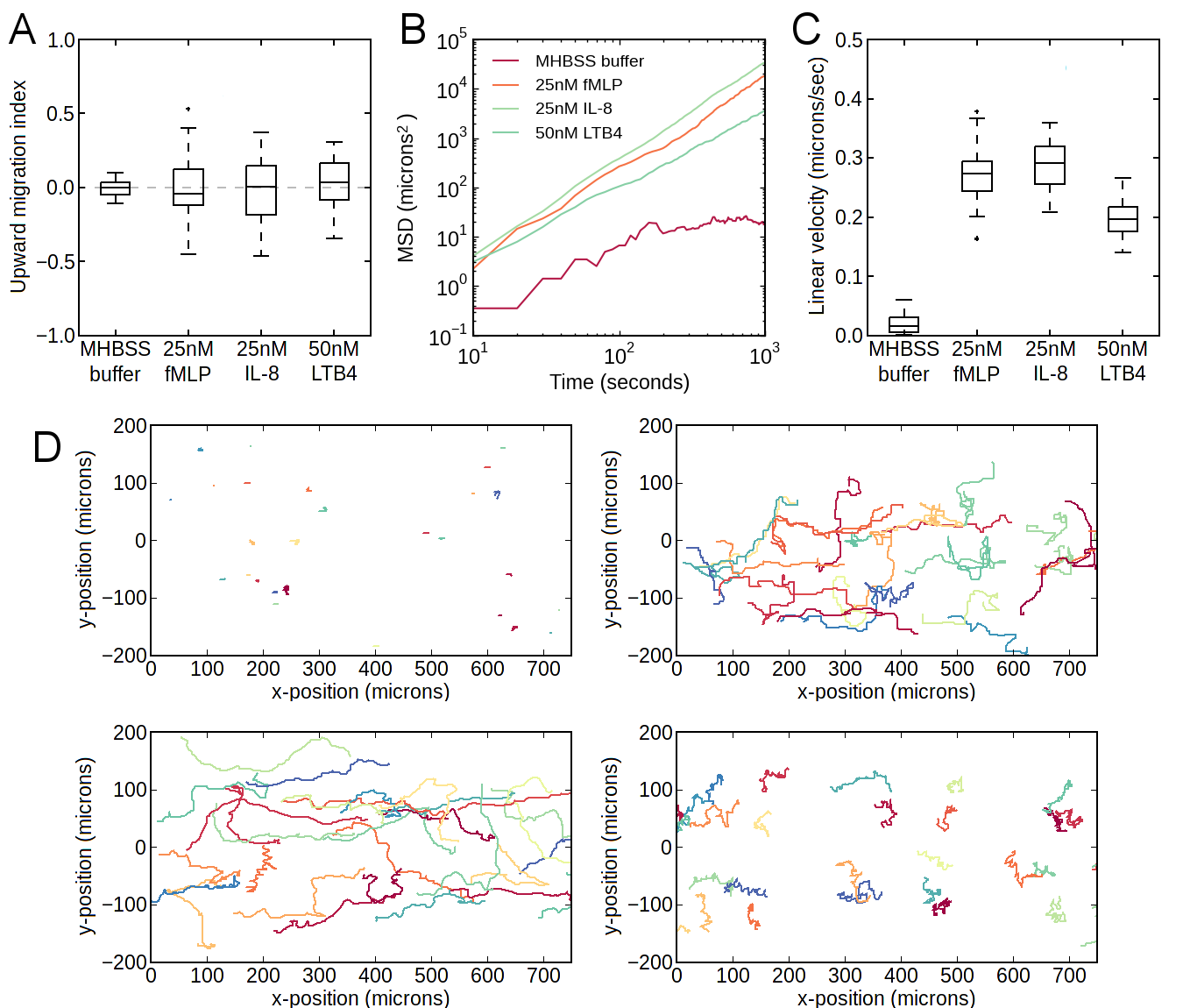

Supplement: Figure S2 — Migration in isotropic attractant conditions. [A] Uniform chemoattractant environments were established by flowing the same solution into both channel inlets. Cells were tracked for 20 minutes in fMLP, IL-8 and LTB4, and the upward migration indices of 30 cells are shown here for comparison against the control (MHBSS buffer only). [B] The mean square displacements (MSD) of the cells from the previous figure as a function of time. Cells were exposed to uniform concentrations of fMLP, IL-8 and LTB4. [C] The average linear velocities of the cells from the previous figure. Again, cells were exposed to uniform concentrations of fMLP, IL-8 and LTB4. [D] Sample trajectories from the previous control experiments. [top left] MHBSS buffer only; [top right] 25 nM fMLP; [bottom left] 25 nM IL-8; [bottom right] 50 nM LTB4. (TIF) [file pone.0085726.s002.tif]

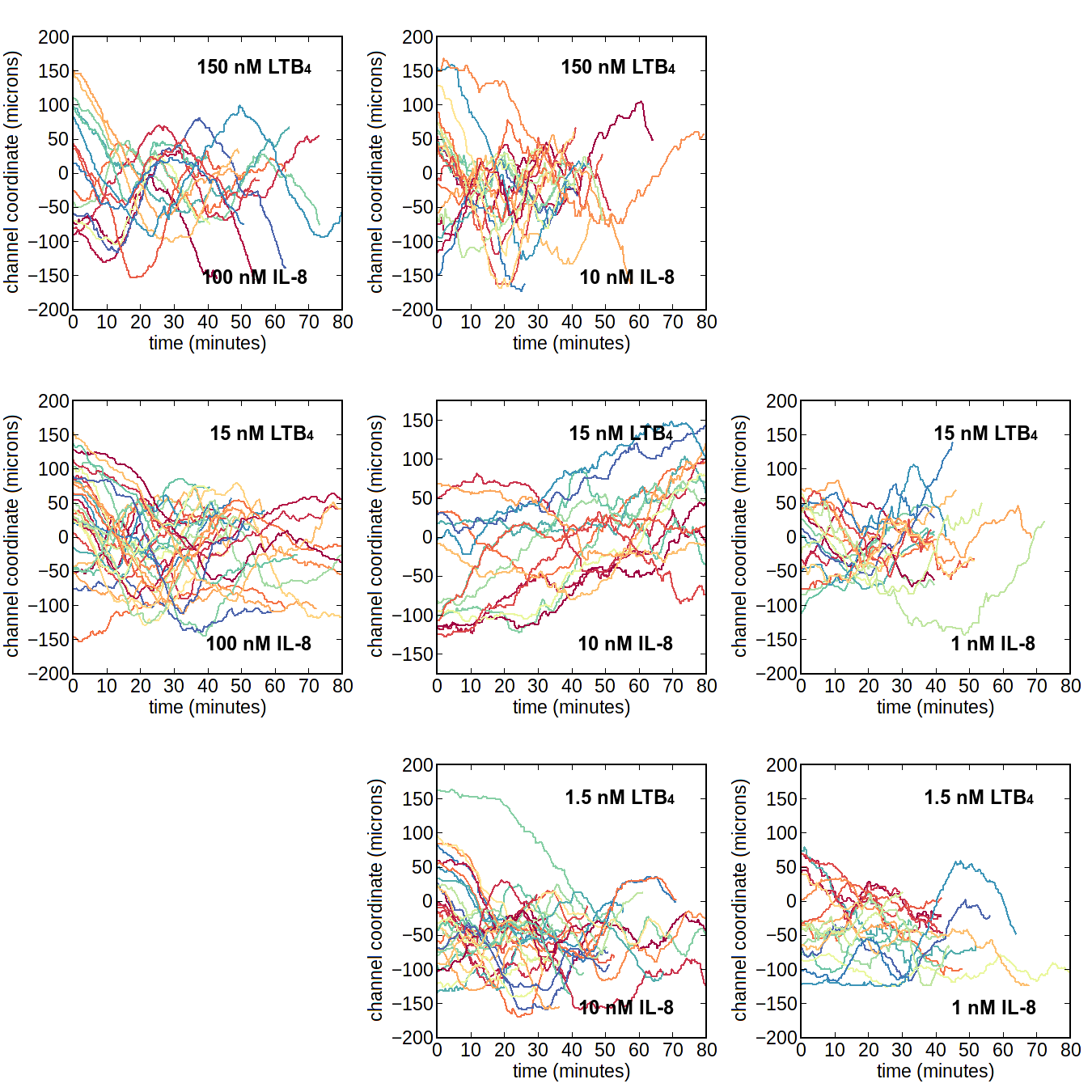

Supplement: Figure S3 — Sample cell trajectories in dual opposing intermediate chemoattractant gradients. Representative cell trajectories indicating the migration behavior of cells in dual opposing gradients of IL-8 and LTB4. (TIF) [file pone.0085726.s003.tif]

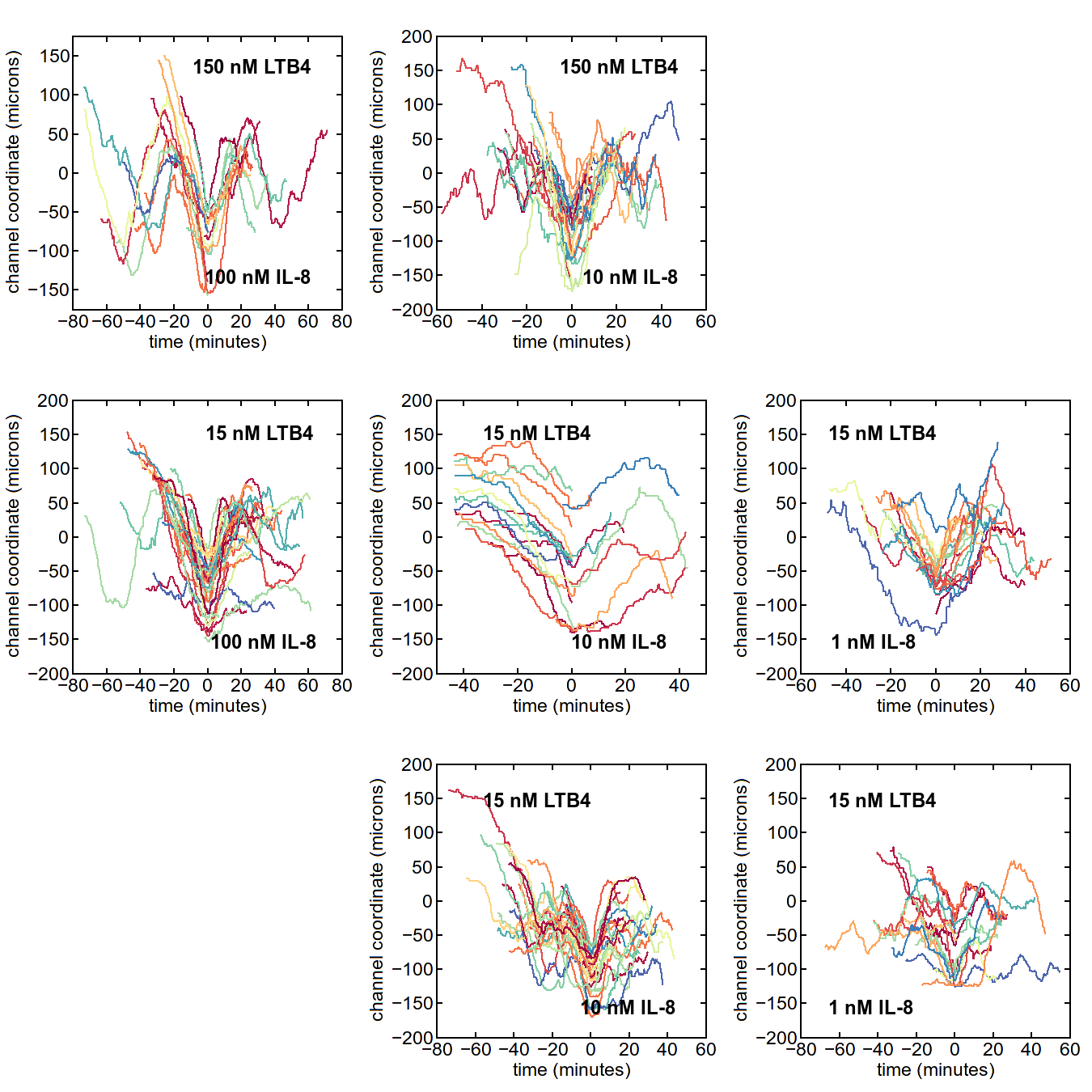

Supplement: Figure S4 — Aligned sample trajectories in dual opposing intermediate chemoattractant gradients. As a visual guide, the cell trajectories from Figure S3 were aligned based on the farthest each cell migrated towards the IL-8 source (denoted zero time). (TIF) [file pone.0085726.s004.tif]

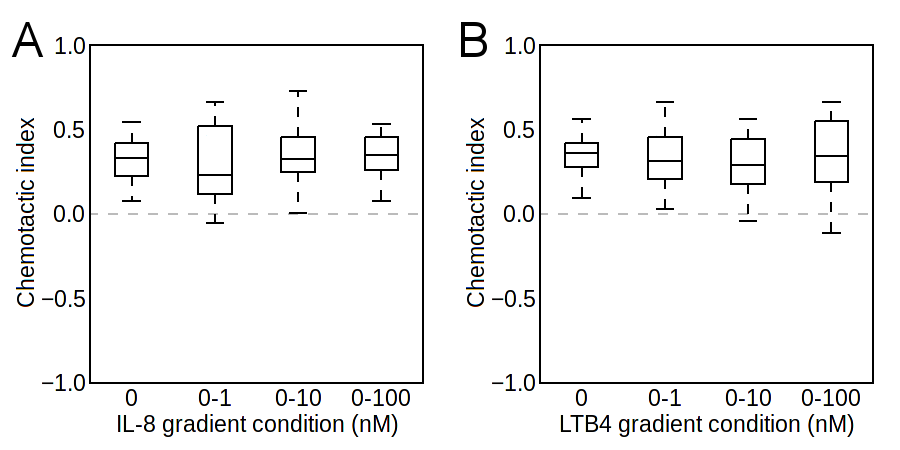

Supplement: Figure S5 — Chemotaxis in opposing linear chemoattractant gradients with fMLP. Chemotactic index in a 0–25 nM fMLP gradient versus varying IL-8 and LTB4 gradients. The fMLP gradient was fixed for all conditions, while the intermediate attractant gradient was varied from no gradient to 0–100 nM for both IL-8 and LTB4. 30 cells were tracked for 40 minutes for each experiment. The correlation with the intermediate chemoattractant gradient was weak with Pearson correlations (r. −0.1694; P. 0.1331) and (r. −0.1304; P. 0.1785), respectively. (TIF) [file pone.0085726.s005.tif]

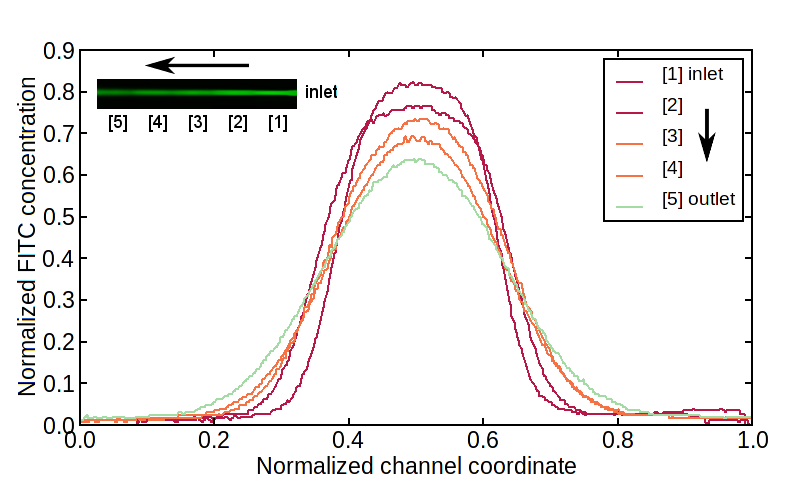

Supplement: Figure S6 — Cross-sectional concentration profile for hill-type gradient. Gradient formation was verified by feeding a fluorescein-labeled solution into the central inlet and an unlabeled solution into the outer inlets. The normalized FITC concentration across the channel cross-section is shown. (TIF) [file pone.0085726.s006.tif]

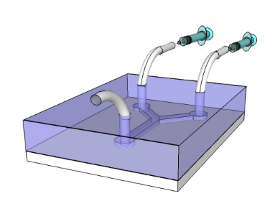

Supplement: Figure S7 — Schematic of microfluidic platform with Y-shaped channel. The platform was comprised of a molded PDMS slab embossed with microchannels and bonded to a glass coverslip. (TIF) [file pone.0085726.s007.tif]

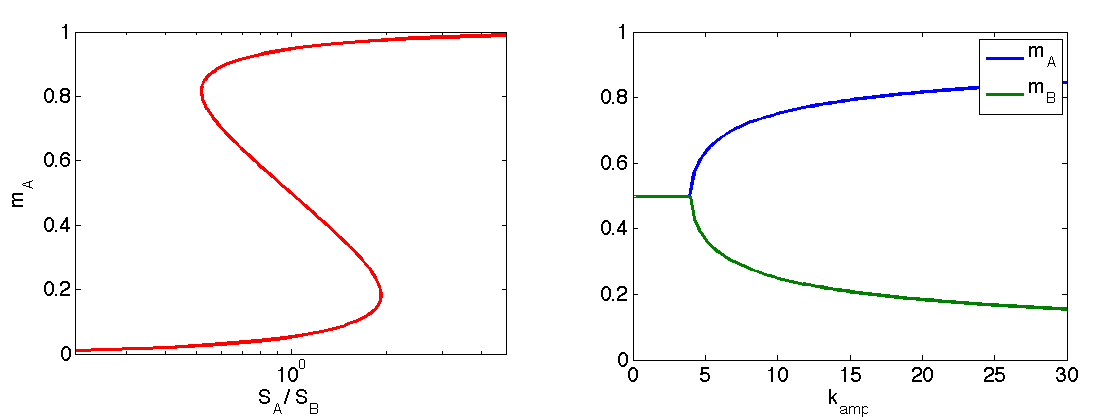

Supplement: Figure S8 — The signaling threshold mechanism. [Left] Response as function of and (). Note the hysteresis in the response – this is necessary to generate the oscillations that results from the overshoot inherent in this mechanism. [Right] Response as a function of amplification gain parameter when . (TIF) [file pone.0085726.s008.tif]

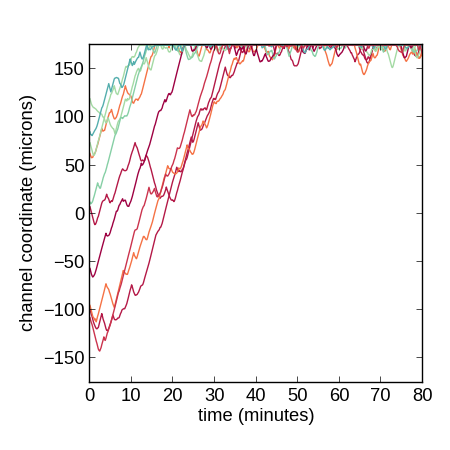

Supplement: Figure S9 — Simulation of the model in a competing gradient of an end-target chemoattractant and intermediate chemoattractant. The parameter values used in this simulation are: µm/s, M, M, , , M (end-target), and M (intermediate). (TIF) [file pone.0085726.s009.tif]

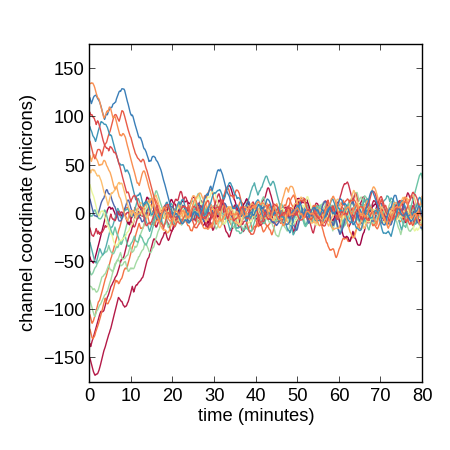

Supplement: Figure S10 — No oscillations are observed in the absence of pseudopod memory. In these simulations, and are fixed at the value 1.0. The parameter values used in this simulation are: µm/s, M, M, and M. (TIF) [file pone.0085726.s010.tif]

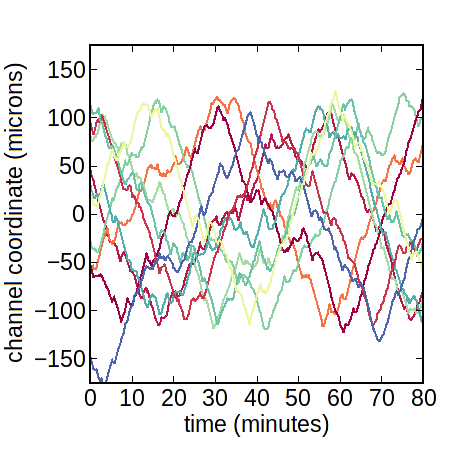

Supplement: Figure S11 — The model is able to robustly generate sustained oscillatory behavior. The initial position of the cells does not affect the amplitude of the oscillatory motion. The parameter values used in this simulation are: µm/s, M, ,M, and M. (TIF) [file pone.0085726.s011.tif]
