# Supplementary material for: Oscillatory Behavior of Neutrophils under Opposing Chemoattractant Gradients Supports a Winner-Take-All Mechanism
Source: PLoS One. 2014 Jan 21;9(1):e85726. doi: 10.1371/journal.pone.0085726 (PMC3897492; doi:10.1371/journal.pone.0085726)
Supplement: Text S1 — Analysis of alternate chemotaxis models. (PDF) [file pone.0085726.s012.pdf]

SUPPORTING INFORMATION
Oscillatory behavior of neutrophils under opposing chemoattractant gradients supports a winner-take-all mechanism

Matthew B. Byrne, Yuki Kimura, Ashish Kapoor, Yuan Heb, Kewin Mattam, Katherine Hasan, Luke N. Olson, Fei Wang, Paul J.A. Kenis, Christopher V. Rao

1 Analysis of alternate chemotaxis models

Foxman *et al.* [1] proposed that the preferential migration of cells toward distant chemokine sources may be explained by sensory adaptation at the receptor level. This hypothesis was further tested in the computational work by Wu *et al.* [2], who showed the sensory adaptation could explain the cell migration patterns observed by Foxman *et al.*. We conducted a broader examination of different chemotaxis models and compared them to our experimental results. As we demonstrate, a mechanism based on sensory adaptation alone will not generate sustained and robust oscillations.

1.1 Baseline model for chemotaxis

We first consider a simple model of the chemotaxis towards a single chemoattractant in one dimension where there is no receptor desensitization or internalization. In this model, we assume that cells move in the direction where the number of bound receptors on their surface C is greatest. In one spatial dimension, we can represent this mathematically as

$$\frac{dx}{dt} \propto \frac{\partial C}{\partial x},$$

where x denotes the position of the cell and t time. Implicit in this model is the assumption that cells move in proportion to the gradient $\partial C/\partial x$. As cells can move only so fast, we assume that

$$\frac{dx}{dt} = v\frac{\chi|S|}{1+\chi|S|}\text{sign}(S)$$

where v is the maximal velocity, χ the chemotaxis coefficient, and $S \triangleq \frac{dC}{dx}$. This form is chosen so that the velocity will saturate when S is large. The reason we take the absolute value of S is to account for negative values or left-ward movement. Note that when S is small,

$$\frac{dx}{dt} \approx v\chi S.$$

To determine C and S, we assume that receptor-ligand binding is described by the following reaction

$$R + L \underset{k_{off}}{\overset{k_{on}}{\rightleftharpoons}} C,$$

where L denotes the attractant and R the receptor. If we assume mass action kinetics and fixed number of total receptors R_{tot}, then C is determined by the differential equation

$$\frac{dC}{dt} = k_{on}L(R_{tot} - C) - k_{off}C.$$

Using this differential equation, we can determine S as follows:

$$\begin{aligned}
\frac{dS}{dt} &= \frac{d}{dt}\left(\frac{\partial C}{\partial x}\right), \\
&= \frac{\partial}{\partial x}\left(\frac{dC}{dt}\right), \\
&= \frac{\partial}{\partial x}\left(k_{on}L(R_{tot} - C) - k_{off}C\right), \\
&= k_{on}(R_{tot} - C)\frac{dL}{dx} - (k_{on}L + k_{off})\frac{dC}{dx}, \\
&= k_{on}(R_{tot} - C)\frac{dL}{dx} - (k_{on}L + k_{off})S.
\end{aligned}$$

We can extend this model to include two different types of attractants and receptors, yielding

$$\begin{aligned}
\frac{d}{dt}\begin{bmatrix} C_A \\ C_B \end{bmatrix} &= \begin{bmatrix} k_{on,A}L_A(R_{tot} - C_A) - k_{off,A}C_A \\ k_{on,B}L_B(R_{tot} - C_B) - k_{off,B}C_B \end{bmatrix} \\
\frac{d}{dt}\begin{bmatrix} S_A \\ S_B \end{bmatrix} &= \begin{bmatrix} k_{on,A}(R_{tot} - C_A)\frac{dL_A}{dx} - (k_{on,A}L_A + k_{off,A})S_A \\ k_{on,B}(R_{tot} - C_B)\frac{dL_B}{dx} - (k_{on,B}L_B + k_{off,B})S_B \end{bmatrix} \\
\frac{dx}{dt} &= \frac{v\chi(S_A + S_B)}{1 + \chi(S_A + S_B)} \approx v\chi(S_A + S_B) \qquad \text{(shallow gradients)}
\end{aligned}$$

where the subscripts A and B denote the specific attractant and cognate receptor. Note that the model assumes that cells employ a vector-sum mechanism where it moves in the direction determined by the sum of the two gradients, where the two attractants are weighted equally. This system of equations can also be nondimensionalized by substituting

$$\bar{C} = \frac{C}{R_{tot}}, \qquad \bar{L} = \frac{L}{k_D}, \qquad \tau = k_{off}t, \qquad \bar{S} = \frac{\sigma S}{R_{tot}},$$
$$\bar{\chi} = \frac{\chi_0 R_{tot}}{v/k_{off}}, \qquad \bar{x} = \frac{x}{\sigma}, \qquad \bar{v} = \frac{v}{\sigma k_{off}}.$$

where σ is the characteristic length, such as the separation between the two chemoattractant sources, and $k_D \triangleq \frac{k_{off}}{k_{on}}$. We can also aggregate these characteristic parameters with a new dimensionless variable

$$\alpha = \frac{vR_{tot}\chi_0}{\sigma^2 k_{off}}.$$

This yields the new dimensionless system of equations:

$$\begin{aligned}
\frac{d}{d\tau}\begin{bmatrix}\bar{C}_A\\ \bar{C}_B\end{bmatrix} &= \begin{bmatrix}\bar{L}_A(1-\bar{C}_A)-\bar{C}_A\\ \bar{L}_B(1-\bar{C}_B)-\bar{C}_B\end{bmatrix}\\
\frac{d}{d\tau}\begin{bmatrix}\bar{S}_A\\ \bar{S}_B\end{bmatrix} &= \begin{bmatrix}(1-\bar{C}_A)\frac{d\bar{L}_A}{d\bar{x}}-(1+\bar{L}_A)\bar{S}_A\\ (1-\bar{C}_B)\frac{d\bar{L}_B}{d\bar{x}}-(1+\bar{L}_B)\bar{S}_B\end{bmatrix}\\
\frac{d\bar{x}}{d\tau} &= \alpha(\bar{S}_A+\bar{S}_B).
\end{aligned}$$

If we then assume that the two chemokines form opposing linear concentration gradients, for instance:

$$\begin{aligned}
L_A(x) &= T_A\left(-\frac{x}{x_s}\right)\\
L_B(x) &= \frac{T_B}{x_s}x
\end{aligned}$$

we can insert the corresponding expressions into $\bar{L}_i$ and $\frac{d\bar{L}_i}{d\bar{x}}$, and solve this system of equations using an ODE solver.

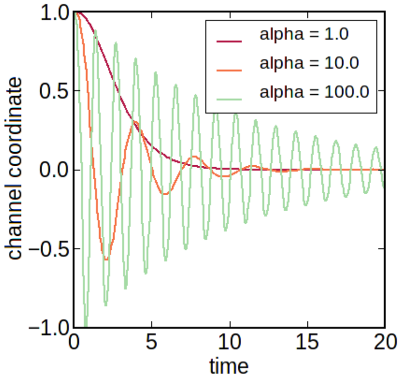

Figure A1: $\alpha > 1$ gives rise to dampened oscillations around the stable fixed point, corresponding to the channel midpoint where the two linear gradients intersect.

From the solution, we note that $\alpha > 1$ gives rise to damped oscillation around the stable steady state, while smaller α leads to monotonic convergence. This suggests that the rate of the receptor kinetics, relative to the speed of cell motion, dictates the asymptotic behavior of the cell (i.e. whether it oscillates). Overall, we find that this simple model is unable to produce sustained oscillatory behavior observered in our experiments. Moreover, the frequency of oscillations vary significantly as the parameter α is modified.

1.2 Receptor internalization

We next add an additional layer of complexity to the model by introducing an intermediate state that accounts for receptor internalization.

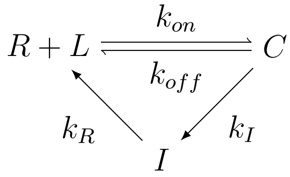

where k_I and k_R represent the rate of receptor internalization and the recycling rate, respectively. If we again assume mass action kinetics, then this reaction scheme is described by the differential equations

$$
\begin{aligned}
\frac{dC}{dt} &= k_{on}L(R_{tot} - C - I) - k_{off}C - k_IC \\
\frac{dI}{dt} &= k_IC - k_RI \\
\frac{dS}{dt} &= k_{on}(R_{tot} - C - I)\frac{dL}{dx} - (k_{on}L + k_{off} + k_I)S
\end{aligned}
$$

For a two receptor model, the governing equations are

$$
\begin{aligned}
\frac{d}{dt}\begin{bmatrix} C_A \\ C_B \end{bmatrix} &= \begin{bmatrix} k_{on,A}L_A(R_{tot} - C_A - I_A) - k_{off,A}C_A - k_{I,A}C_A \\ k_{on,B}L_B(R_{tot} - C_B - I_B) - k_{off,B}C_B - k_{I,B}C_B \end{bmatrix} \\
\frac{d}{dt}\begin{bmatrix} I_A \\ I_B \end{bmatrix} &= \begin{bmatrix} k_{I,A}C_A - k_{R,A}I_A \\ k_{I,B}C_B - k_{R,B}I_B \end{bmatrix} \\
\frac{d}{dt}\begin{bmatrix} S_A \\ S_B \end{bmatrix} &= \begin{bmatrix} k_{on,A}(R_{tot} - C_A - I_A)\frac{dL_A}{dx} - (k_{on,A}L_A + k_{off,A} + k_{I,A})S_A \\ k_{on,B}(R_{tot} - C_B - I_B)\frac{dL_B}{dx} - (k_{on,B}L_B + k_{off,B} + k_{I,B})S_B \end{bmatrix} \\
\frac{dx}{dt} &\approx v\chi_0(S_A + S_B) \qquad \text{(shallow gradients)}
\end{aligned}
$$

As before, we can nondimensionalize the equations using the definitions

$$
\begin{aligned}
&\bar{C} = \frac{C}{R_{tot}}, \qquad \bar{L} = \frac{L}{k_D}, \qquad \tau = k_{off}t, \qquad \bar{S} = \frac{\sigma S}{R_{tot}}, \qquad \bar{k}_I = \frac{k_I}{k_{off}}, \\
&\bar{I} = \frac{I}{R_{tot}}, \qquad \bar{\chi} = \frac{\chi_0 R_{tot}}{v/k_{off}}, \qquad \bar{x} = \frac{x}{\sigma}, \qquad \bar{v} = \frac{v}{\sigma k_{off}}, \qquad \bar{k}_R = \frac{k_R}{k_{off}}, \\
&\alpha = \frac{vR_{tot}\chi_0}{\sigma^2 k_{off}}.
\end{aligned}
$$

and obtain the dimensionless set of equations:

$$
\begin{aligned}
\frac{d}{d\tau}\begin{bmatrix}\bar{C_A}\\ \bar{C_B}\end{bmatrix} &= \begin{bmatrix}L_A(1-\bar{C_A}-\bar{I_A})-\bar{C_A}-\bar{k}_{I,A}\bar{C_A}\\ L_B(1-\bar{C_B}-\bar{I_B})-\bar{C_B}-\bar{k}_{I,B}\bar{C_B}\end{bmatrix}\\
\frac{d}{d\tau}\begin{bmatrix}\bar{I_A}\\ \bar{I_B}\end{bmatrix} &= \begin{bmatrix}\bar{k}_{I,A}\bar{C_A}-\bar{k}_{R,A}\bar{I_A}\\ \bar{k}_{I,B}\bar{C_B}-\bar{k}_{R,B}\bar{I_B}\end{bmatrix}\\
\frac{d}{d\tau}\begin{bmatrix}\bar{S_A}\\ \bar{S_B}\end{bmatrix} &= \begin{bmatrix}(1-\bar{C_A}-\bar{I_A})\frac{d\bar{L_A}}{d\bar{x}}-(1+\bar{L_A}+\bar{k}_{I,A})\bar{S_A}\\ (1-\bar{C_B}-\bar{I_B})\frac{d\bar{L_B}}{d\bar{x}}-(1+\bar{L_B}+\bar{k}_{I,B})\bar{S_B}\end{bmatrix}\\
\frac{dx}{d\tau} &= \alpha(\bar{S_A}+\bar{S_B})
\end{aligned}
$$

Solving this system of equations using an ODE solver, we obtain the following behaviors. Note that due to the introduction of additional variables, we have more degrees of freedom.

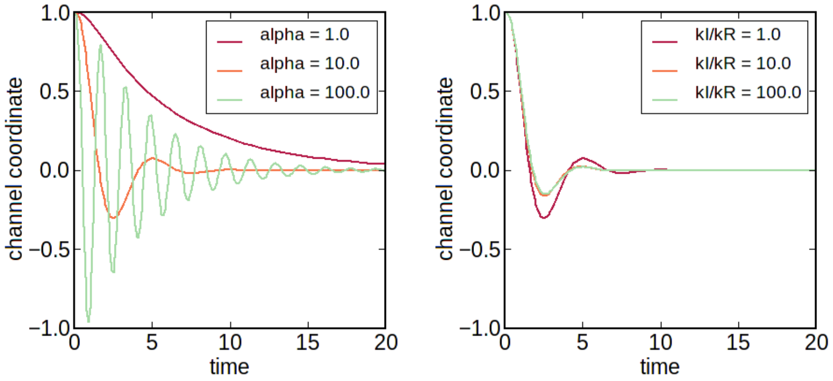

Figure A2: [Left] Fixed $k_{I,A}/k_{R,A} = k_{I,B}/k_{R,B} = 1$. [Right] $\alpha = 10$ is fixed and $k_{I,A}/k_{R,A} = k_{I,B}/k_{R,B}$ is varied.

In this case, we again note that $\alpha > 1$ gives rise to dampened oscillations around the stable fixed point, while $\alpha \leq 1$ leads to monotonic convergence. Increasing α has the effect of increasing frequency. Meanwhile, a larger k_I/k_R gives rise to a smaller amplitude in the damped oscillator.

We might also assume that both the internalized(I) and bound(C) states of the receptor can simultaneously contribute to the chemotactic response. In this instance, we need an additional set of variables to denote the internal gradient of I, or $J = \frac{dI}{dx}$ for the two receptor

types, resulting in the following:

$$
\begin{aligned}
\frac{d}{dt}\begin{bmatrix} C_A \\ C_B \end{bmatrix} &= \begin{bmatrix} k_{on,A}L_A(R_{tot} - C_A - I_A) - k_{off,A}C_A - k_{I,A}C_A \\ k_{on,B}L_B(R_{tot} - C_B - I_B) - k_{off,B}C_B - k_{I,B}C_B \end{bmatrix} \\
\frac{d}{dt}\begin{bmatrix} I_A \\ I_B \end{bmatrix} &= \begin{bmatrix} k_{I,A}C_A - k_{R,A}I_A \\ k_{I,B}C_B - k_{R,B}I_B \end{bmatrix} \\
\frac{d}{dt}\begin{bmatrix} S_A \\ S_B \end{bmatrix} &= \begin{bmatrix} k_{on,A}(R_{tot} - C_A - I_A)\frac{dL_A}{dx} - (k_{on,A}L_A + k_{off,A} + k_{I,A})S_A - k_{on,A}L_AJ_A \\ k_{on,B}(R_{tot} - C_B - I_B)\frac{dL_B}{dx} - (k_{on,B}L_B + k_{off,B} + k_{I,B})S_B - k_{on,B}L_BJ_B \end{bmatrix} \\
\frac{d}{dt}\begin{bmatrix} J_A \\ J_B \end{bmatrix} &= \begin{bmatrix} k_{I,A}S_A - k_{R,A}J_A \\ k_{I,B}S_B - k_{R,B}J_B \end{bmatrix} \\
\frac{dx}{dt} &\approx v\chi_0(S_A + S_B + J_A + J_B) \qquad \text{(shallow gradients)}
\end{aligned}
$$

As before, if we nondimensionalize using the following definitions

$$
\begin{aligned}
&\bar{C} = \frac{C}{R_{tot}}, \qquad \bar{L} = \frac{L}{k_D}, \qquad \tau = k_{off}t, \qquad \bar{S} = \frac{\sigma S}{R_{tot}}, \qquad \bar{k_I} = \frac{k_I}{k_{off}}, \\
&\bar{I} = \frac{I}{R_{tot}}, \qquad \bar{\chi} = \frac{\chi_0 R_{tot}}{v/k_{off}}, \qquad \bar{x} = \frac{x}{\sigma}, \qquad \bar{v} = \frac{v}{\sigma k_{off}}, \qquad \bar{k_R} = \frac{k_R}{k_{off}}, \\
&\alpha = \frac{vR_{tot}\chi_0}{\sigma^2 k_{off}}.
\end{aligned}
$$

and obtain the dimensionless equations

$$
\begin{aligned}
\frac{d}{d\tau}\begin{bmatrix} \bar{C_A} \\ \bar{C_B} \end{bmatrix} &= \begin{bmatrix} L_A(1 - \bar{C_A} - \bar{I_A}) - \bar{C_A} - \bar{k_{I,A}}\bar{C_A} \\ L_B(1 - \bar{C_B} - \bar{I_B}) - \bar{C_B} - \bar{k_{I,B}}\bar{C_B} \end{bmatrix} \\
\frac{d}{d\tau}\begin{bmatrix} \bar{I_A} \\ \bar{I_B} \end{bmatrix} &= \begin{bmatrix} \bar{k_{I,A}}\bar{C_A} - \bar{k_{R,A}}\bar{I_A} \\ \bar{k_{I,B}}\bar{C_B} - \bar{k_{R,B}}\bar{I_B} \end{bmatrix} \\
\frac{d}{d\tau}\begin{bmatrix} \bar{S_A} \\ \bar{S_B} \end{bmatrix} &= \begin{bmatrix} (1 - \bar{C_A} - \bar{I_A})\frac{d\bar{L_A}}{d\bar{x}} - (1 + \bar{L_A} + \bar{k_{I,A}})\bar{S_A} - \bar{L_A}\bar{J_A} \\ (1 - \bar{C_B} - \bar{I_B})\frac{d\bar{L_B}}{d\bar{x}} - (1 + \bar{L_B} + \bar{k_{I,B}})\bar{S_B} - \bar{L_B}\bar{J_B} \end{bmatrix} \\
\frac{d}{d\tau}\begin{bmatrix} \bar{J_A} \\ \bar{J_B} \end{bmatrix} &= \begin{bmatrix} \bar{k_{I,A}}\bar{S_A} - \bar{k_{R,A}}\bar{J_A} \\ \bar{k_{I,B}}\bar{S_B} - \bar{k_{R,B}}\bar{J_B} \end{bmatrix} \\
\frac{dx}{d\tau} &= \alpha(\bar{S_A} + \bar{S_B} + \bar{J_A} + \bar{J_B})
\end{aligned}
$$

Note that this model, however, requires that the internalized receptors retain spatial heterogeneity, which may not be an accurate assumption. Again, we can solve this system using an ODE solver.

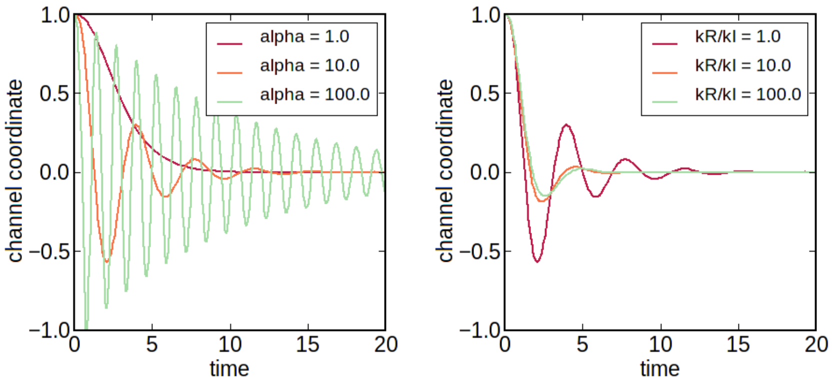

Figure A3: [Left] Fixed $(k_{I,A}, k_{R,A}) = (k_{I,B}, k_{R,B}) = (1,1)$. [Right] $\alpha = 10$ is fixed and $(k_{I,A}, k_{R,A}) = (k_{I,B}, k_{R,B})$ is varied.

From the plots we note that the behavior is fairly similar to the previous case, though the additional signaling from internalized receptors leads to slightly different dynamics. In particular, we can note a stronger overall response and faster cell movement. Otherwise, increasing α still has the effect of increasing frequency, while a larger k_I and k_R still yield a smaller amplitude.

In both models of receptor internalization, the asymptotic behavior is still convergence to the stable fixed point at the center of the channel. The analysis suggests that internalization alone cannot produce a stable limit cycle in a robust manner.

1.3 Receptor desensitization and internalization

For the next example we add an additional desensitized (but not internalized) receptor state to the model to examine how the transient behavior is affected. This network structure was employed by Wu *et al.* to demonstrate preferential migration of cells toward the distant source [2].

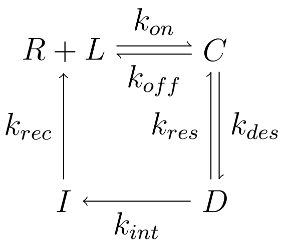

By following a derivation similar to the previous cases, we obtain the following system of equations.

$$
\begin{aligned}
\frac{d}{d\tau}\begin{bmatrix}\bar{C_A}\\ \bar{C_B}\end{bmatrix} &= \begin{bmatrix}L_A(1-\bar{C_A}-\bar{I_A}-\bar{D_A})-(1+k_{des,A})\bar{C_A}+k_{res,A}\bar{D_A}\\ L_B(1-\bar{C_B}-\bar{I_B}-\bar{D_B})-(1+k_{des,B})\bar{C_B}+k_{res,B}\bar{D_B}\end{bmatrix}\\
\frac{d}{d\tau}\begin{bmatrix}\bar{D_A}\\ \bar{D_B}\end{bmatrix} &= \begin{bmatrix}k_{des,A}\bar{C_A}-k_{res,A}\bar{D_A}-k_{I,A}\bar{D_A}\\ k_{des,B}\bar{C_B}-k_{res,B}\bar{D_B}-k_{I,B}\bar{D_B}\end{bmatrix}\\
\frac{d}{d\tau}\begin{bmatrix}\bar{I_A}\\ \bar{I_B}\end{bmatrix} &= \begin{bmatrix}k_{I,A}\bar{D_A}-k_{rec,1}\bar{I_A}\\ k_{I,B}\bar{D_B}-k_{rec,2}\bar{I_B}\end{bmatrix}\\
\frac{d}{d\tau}\begin{bmatrix}\bar{S_A}\\ \bar{S_B}\end{bmatrix} &= \begin{bmatrix}(1-\bar{C_A}-\bar{D_A}-\bar{I_A})\frac{dL_A}{dx}\bar{L_A}-(1+\bar{L_A}+k_{des,A})\bar{S_A}\\ (1-\bar{C_B}-\bar{D_B}-\bar{I_B})\frac{dL_B}{dx}\bar{L_B}-(1+\bar{L_B}+k_{des,B})\bar{S_B}\end{bmatrix}\\
\frac{dx}{d\tau} &= \alpha(\bar{S_A}+\bar{S_B})
\end{aligned}
$$

Note from the results that despite the increase in degrees of freedom, again the asymptotic behavior is that of convergence toward the stable fixed point (at the channel median).

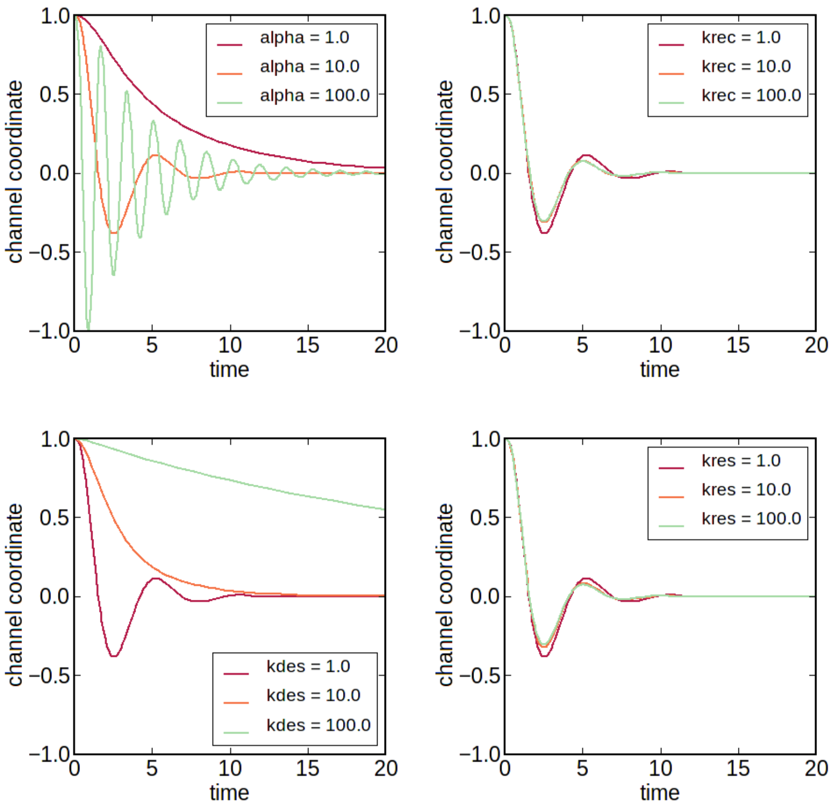

Figure A4: [Top left] Fixed $k_{int} = k_{rec} = k_{des} = k_{res} = 1$, α varied. [Top right] $\alpha = 10$, $k_{int} = k_{des} = k_{res} = 1$ fixed and k_{rec} is varied. [Bottom left] $\alpha = 10$, $k_{int} = k_{rec} = k_{res} = 1$ fixed and k_{des} is varied. [Bottom right] $\alpha = 10$, $k_{int} = k_{rec} = k_{des} = 1$ fixed and k_{res} is varied.

As with the previous models, the asymptotic behavior is that of either monotonic convergence or damped oscillation toward a stable fixed point, in the case of linear gradients. In

their paper, Wu *et al.* used nonlinear gradients defined using a power law. However, while their model predicted preferential migration of cells toward the distant source, it was not evident whether the cells would oscillate back and forth between the two sources as observed in our experiments.

1.4 Internal memory model

It is well understood that in isotropic chemoattractant environments, neutrophils exhibit directional persistence, in which their migrational steps are directionally-correlated on the order of several minutes [3]. This phenomenon could be attributed to a form of internal memory, in which detected signals are amplified and sustained over the same timeframe, with attenuation of signal decay. The next network model applies this concept by adding an additional internal state variable, which serves to provide short-term memory of the current processed signal within the cell. The goal was to see if this approach could lead to sustained oscillatory motion in the linear gradients.

Using the following network model,

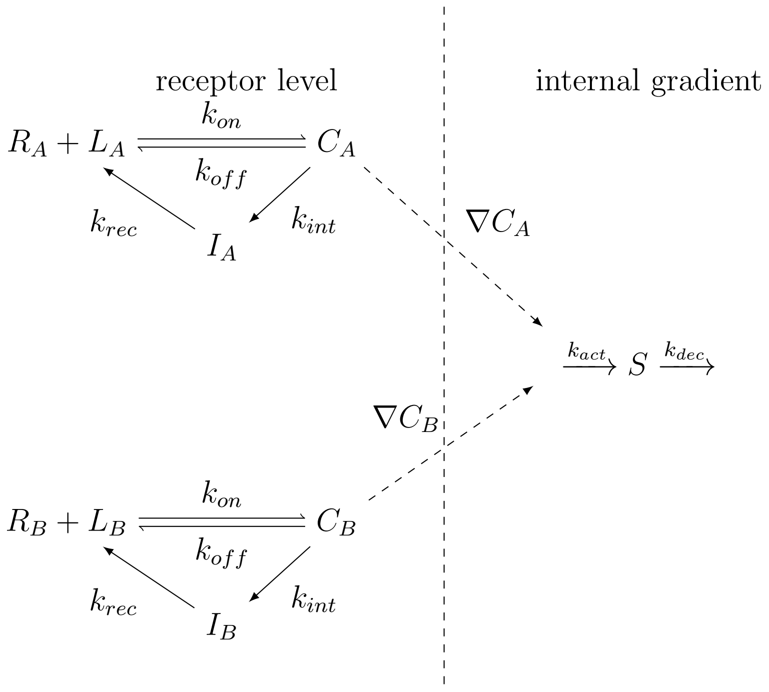

we constructed the corresponding system of equations, where the internal interpreted gradients ∇C_A and ∇C_B were used to influence the state variable through a rate k_{act}.

$$\frac{d}{dt}\begin{bmatrix}C_A\\C_B\end{bmatrix} = \begin{bmatrix}k_{on,A}L_A(R_{tot}-C_A-I_A)-k_{off,A}C_A-k_{int,A}C_A\\k_{on,B}L_B(R_{tot}-C_B-I_B)-k_{off,B}C_B-k_{int,B}C_B\end{bmatrix}$$
$$\frac{d}{dt}\begin{bmatrix}I_A\\I_B\end{bmatrix} = \begin{bmatrix}k_{int,A}C_A-k_{rec,A}I_A\\k_{int,B}C_B-k_{rec,B}I_B\end{bmatrix}$$
$$\frac{d}{dt}\begin{bmatrix}\nabla C_A\\\nabla C_B\end{bmatrix} = \begin{bmatrix}k_{on,A}(R_{tot}-C_A-I_A)\frac{dL_A}{dx}-(k_{on,A}L_A+k_{off,A}+k_{int,A})\nabla C_A\\k_{on,B}(R_{tot}-C_B-I_B)\frac{dL_B}{dx}-(k_{on,B}L_B+k_{off,B}+k_{int,B})\nabla C_B\end{bmatrix}$$
$$\frac{dS}{dt} = k_{act}(\nabla C_A+\nabla C_B)-k_{dec}S$$
$$\frac{dx}{dt} \approx \alpha S$$

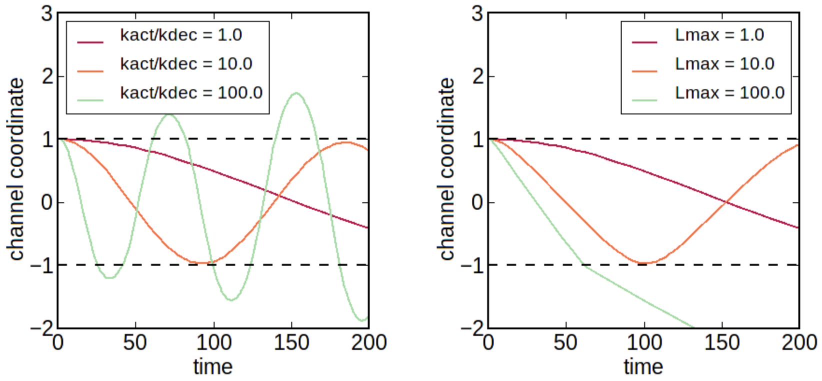

Figure A5: [Left] Fixed $\alpha = 1$ and let k_{act}/k_{dec} vary. However, fixing k_{act}/k_{dec} and changing α has a similar effect. In either case, controlling the amplitude of the oscillation also affects the frequency. [Right] The effect of changing the concentration of both chemoattractant maxima with other parameters unchanged.

We can observe from the equations that modifying the parameter α and the ratio k_{act}/k_{dec} are tightly related - increasing either of them appears to enlarge both the frequency and the amplitude of the response. From the simulations, it was also evident that this system was particularly sensitive to parameter changes, and in some cases, where the activation of the signal was significantly faster than the decay rate, the system became unstable. The application of realistic parameter choices from the literature resulted in similarly sensitive results, with minor changes in the source concentration resulting in vastly different behaviors. This high sensitivity appears to be incompatible with the robust oscillatory response that was observed over a wide range of conditions.

1.5 Oelz-Schmeiser-Soreff (OSS) model

In contrast to the network-based kinetics models, Oelz *et al.* [4] used a relaxation model to argue their case. They demonstrated that cells can in fact undergo preferential migration to the distant source, under the simple condition that their sensitivities do not adapt

immediately to the local concentrations. This was the first model to predict, under certain parameter ranges, that cells might exhibit oscillatory dynamics between two opposing chemokine gradients. The basis of their argument was that a Hopf bifurcation around the steady state could lead to a stable limit cycle, and hence show sustained oscillation in the cell trajectory.

Their model took the following form (considering only the deterministic dynamics and using slightly different nomenclature):

$$\begin{aligned}\dot{x} &= (S_A \nabla L_A + S_B \nabla L_B)\\ \dot{S_A} &= \alpha_1(\hat{\chi_1}(L_A) - S_A)\\ \dot{S_B} &= \alpha_2(\hat{\chi_2}(L_B) - S_B)\end{aligned}$$

where α was defined as the rate of adaptation of the sensitivity to its target value. $\hat{\chi}$ was defined as:

$$\hat{\chi_i}(L) = \chi_i^{min} + \frac{1}{(AL)_i + \frac{1}{\chi_i^{max} - \chi_i^{min}}}.$$

In their analysis, they then applied the parameters $A = I$, $\alpha_i = 1$, $S_{min} = 0$ and $S_{max} = \infty$, which simplified the model to

$$\begin{aligned}\dot{x} &= \frac{1}{\epsilon}(S_A \nabla L_A + S_B \nabla L_B)\\ \dot{S_A} &= \frac{1}{L_A} - S_A\\ \dot{S_B} &= \frac{1}{L_B} - S_B\end{aligned}$$

Using concentrations in the form of Gaussian point sources centered at x_i with spread T_i,

$$L_i(x) = \exp\left(\frac{-|x - x_i|^2}{T_i}\right).$$

they then showed that the single critical point of the system is given by

$$\begin{pmatrix} x_\infty \\ y_\infty \\ S_{1\infty} \\ S_{2\infty} \end{pmatrix} = \begin{pmatrix} \frac{x_1 T_B + x_2 T_A}{T_A + T_B} \\ 0 \\ 1/L_A \\ 1/L_B \end{pmatrix}.$$

The eigenvalues of the linearized system around this point are

$$\lambda_1 = -\frac{2(T_A + T_B)}{\epsilon T_A T_B}, \qquad \lambda_2 = -1,$$

$$\lambda_3 = -\frac{p}{2} + \sqrt{\frac{p^2}{4} - q}, \qquad \lambda_4 = -\frac{p}{2} - \sqrt{\frac{p^2}{4} - q},$$

where

$$p = \frac{2(T_A + T_B)}{\epsilon T_A T_B} - \frac{8(x_2 - x_1)^2}{\epsilon (T_A + T_B)^2} + 1 \qquad \text{and} \qquad q = \frac{2(T_A + T_B)}{\epsilon T_A T_B}.$$

Since the complex conjugate pair λ_3 and λ_4 cross the imaginary axis when $q > \frac{p^2}{4}$, a Hopf bifurcation is observed (when ϵ satisfies $p < 0$). We can also confirm this through simulation.

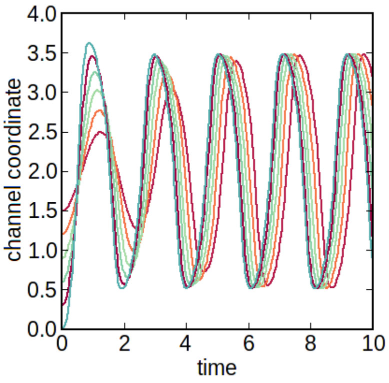

Figure A6: Cell trajectories in Gaussian gradients located at $x = 0$ and $x = 4$, with varying initial positions.

The key to this argument, however, is the Gaussian concentration profile used in the analysis. In particular, our experimental results suggest that cells can exhibit sustained oscillatory motion even in the presence of opposing linear gradients. However, we can show that the choice of linear gradients can lead to different asymptotic behavior.

Let $x_A = 0$ and $x_B = x_s$ be the position of the two chemokine maxima, where we define the opposing linear concentration gradients by

$$\begin{aligned} L_A(x) &= T_A - \frac{T_A}{x_s}x \\ L_B(x) &= \frac{T_B}{x_s}x \end{aligned}$$

Plugging this new concentration profile into the model (and omitting y due to symmetry), we find that the critical point of the system is now given by

$$\begin{pmatrix} x_\infty \\ S_{1\infty} \\ S_{2\infty} \end{pmatrix} = \begin{pmatrix} \frac{x_s}{2} \\ 1/L_A \\ 1/L_B \end{pmatrix}$$

regardless of the choice of T_A and T_B. The eigenvalues of the linearized system around this point are then

$$\lambda_1 = -1, \qquad \lambda_2 = -\frac{1}{2} - \frac{\sqrt{\epsilon x_s^4 - 32x_s^2}}{2\sqrt{\epsilon}x_s^2}, \qquad \lambda_3 = -\frac{1}{2} + \frac{\sqrt{\epsilon x_s^4 - 32x_s^2}}{2\sqrt{\epsilon}x_s^2}.$$

Note that in this case, the real parts of all eigenvalues are always negative, implying asymptotic convergence to the stable fixed point. Moreover, λ_2 and λ_3 become complex when $(\epsilon x_s^4 - 32x_s^2) < 0$ or

$$\frac{1}{\epsilon} < \frac{x_s^2}{32},$$

which gives us the condition for damped oscillation as opposed to monotonic convergence. This can also be demonstrated via simulation as shown below. One may note that the amplitude of the damped oscillation is dependent on the distance between the initial position and the stable fixed point, contrary to what was observed experimentally. Our results showed that the neutrophils were strongly robust against variation in the initial conditions, leading to comparable amplitudes in the oscillatory response.

Furthermore, the sustained oscillations observed in the Oelz model under opposing gaussian gradients was a result of Hopf bifurcation and a particular selection of parameters. It remains unclear whether this mechanism would be sufficiently robust against the varying chemoattractant conditions to which we exposed our cells experimentally. We also find that in the linear gradient case, the OSS model exhibits asymptotic convergence to a stable fixed point under all parameter choices.

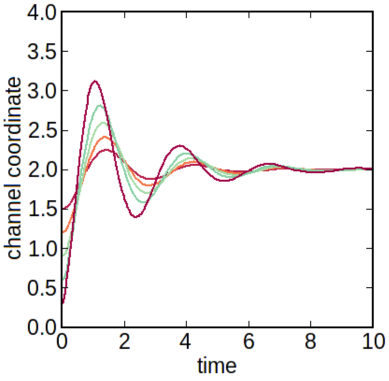

Figure A7: Cell trajectories in dual opposing linear gradients between $x = 0$ and $x = 4$, with varying initial positions. Note the damped oscillatory behavior instead of the stable limit cycle observed under dual Gaussian gradients.

1.6 Feedback-based model

We consider here a simplified version of the feedback-based model discussed in the main text. The goal is to separate the feedback-based component of the model from the pseudopod-based component. As we demonstrate, the latter is not necessary for oscillatory motion – rather, only the feedback-based component is.

If we assume deterministic dynamics, then a simplified version of the feedback-based model can be described by the following set of differential equations:

$$\dot{x} = \alpha \left(m_A \nabla L_A + m_B \nabla L_B \right)$$
$$\dot{m}_A = \epsilon \frac{f_A}{f_A + f_B} - m_A$$
$$\dot{m}_B = \epsilon \frac{f_B}{f_B + f_B} - m_B$$

where

$$f_A = \chi_A \left(1 + k_{amp} m_A^2\right) \text{ and } f_B = \chi_B \left(1 + k_{amp} m_B^2\right)$$

and

$$\chi_A = \frac{1}{1+L_A} \quad \text{and} \quad \chi_B = \frac{1}{1+L_B}.$$

For simplicity, we have cast this equation in dimensionless form. The parameter α is the dimensionless chemotaxis coefficient and the parameter ϵ provides the relative time scales for the cell motion and the feedback loops governing the weights m_A and m_B.

As can be seen from the figure below, this simple model generates oscillatory motion that is robust to the initial positions.

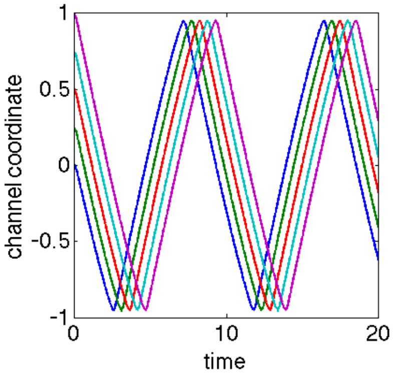

Figure A8: Cell trajectories in dual opposing linear gradients between $x = -1$ and $x = 1$, with varying initial positions. The parameter used for the simulation are: $\alpha = 1$, $\epsilon = 20$, and $k_{amp} = 20$.

The parameter α determines the period of these oscillations and the parameter k_{amp} the amplitude. The later can also be seen from the magnitude of the threshold shown **Figure S7**. The only requirement of oscillations is that the parameter ϵ be sufficiently large relative to α. In other words, the positive feedback loop must operate on a fast time scale than cell motion, otherwise the oscillations will not occur.

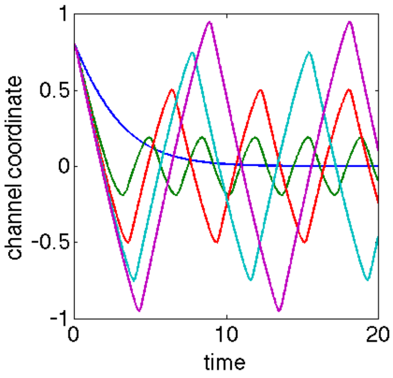

Figure A9: Cell trajectories in dual opposing linear gradients between $x = -1$ and $x = 1$ for varying values of the parameter k_{amp} (ranging from 1 to 20). The parameter used for the simulation are: $\alpha = 1$ and $\epsilon = 20$. Note the amplitude increases as k_{amp} increases.

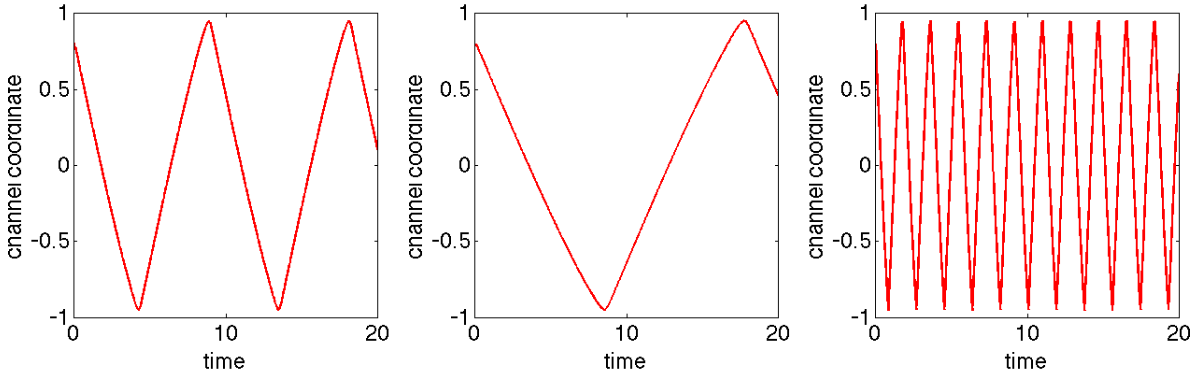

Figure A10: Cell trajectories in dual opposing linear gradients between $x = -1$ and $x = 1$ for varying values of the parameter α (Left: 0.5, Middle: 1, and Right: 5). The parameter used for the simulation are: $\epsilon = 20\alpha$ and $k_{amp} = 20$.

References

[1] EF Foxman, EJ Kunkel, and EC Butcher. Integrating conflicting chemotactic signals: The role of memory in leukocyte navigation. *J Cell Biol*, 147:577–588, 1999.

[2] L Wu and F Lin. Modeling cell gradient sensing and migration in competing chemoattractant fields. *PLoS ONE*, 6(4), 2011.

[3] EF Foxman, JJ Campbell, and EC Butcher. Multistep navigation and the combinatorial control of leukocyte chemotaxis. *J Cell Biol*, 139:1349–1360, 1997.

[4] D Oelz, C Schmeiser, and A Soreff. Multistep navigation of leukocytes: a stochastic model with memory effects. *Math Med Biol*, 22(4):291–303, 2005.
